# Biofunctional Testing of a Degradable Implant Made by Mg-Nd and Mg-Zn Alloys Used for Bone Defects

**DOI:** 10.3390/biomimetics11030169

**Published:** 2026-03-02

**Authors:** Veronica Manescu (Paltanea), Aurora Antoniac, Gheorghe Paltanea, Iulian Antoniac, Emöke Páll, Maria Cristina Moraru, Alexandra Iulia Dreanca, Bogdan Sevastre, Radu Stefanoiu, Robert Ciocoiu, Sebastian Gradinaru, Julietta V. Rau, Marius Manole

**Affiliations:** 1 Faculty of Material Science and Engineering, National University of Science and Technology Politehnica Bucharest, 313 Splaiul Independentei, District 6, RO-060042 Bucharest, Romaniaantoniac.iulian@gmail.com (I.A.); radu.stefanoiu@upb.ro (R.S.); robert.ciocoiu@upb.ro (R.C.); 2 Faculty of Electrical Engineering, National University of Science and Technology Politehnica Bucharest, 313 Splaiul Independentei, District 6, RO-060042 Bucharest, Romania; gheorghe.paltanea@upb.ro (G.P.); 3 Academy of Romanian Scientists, 54 Splaiul Independentei, RO-050094 Bucharest, Romania; 4 Faculty of Veterinary Medicine, University of Agricultural Sciences and Veterinary Medicine of Cluj-Napoca, 3-5 Calea Manastur, RO-400372 Cluj-Napoca, Romania; emoke.pall@usamvcluj.ro (E.P.); maria.moraru@usamvcluj.ro (M.C.M.); alexandra.dreanca@usamvcluj.ro (A.I.D.); bogdan.sevastre@usamvcluj.ro (B.S.); 5 Department of Medical-Clinical Discipline, Faculty of Medicine, Titu Maiorescu University, 67A Gheorghe Petrascu, RO-031593 Bucharest, Romania; sebastian.gradinaru@prof.utm.ro (S.G.); 6 Department of General Surgery, County Hospital Ilfov, RO-050474 Bucharest, Romania; 7 Istituto di Struttura della Materia, Consiglio Nazionale delle Ricerche (ISM-CNR), Via del Fosso del Cavaliere 100, 00133 Rome, Italy; giulietta.rau@ism.cnr.it (J.V.R.); 8 Department of Analytical, Physical and Colloid Chemistry, Institute of Pharmacy, I.M. Sechenov First Moscow State Medical University, Trubetskaya St. 8, build. 2, 119048 Moscow, Russia; 9 Department of Prosthetics and Dental Materials, Faculty of Dentistry, Iuliu Hatieganu University of Medicine and Pharmacy, 8 Victor Babes, RO-400012 Cluj-Napoca, Romania; marius.manole@umfcluj.ro (M.M.)

**Keywords:** magnesium-based implants, mechanical attributes, immersion tests, cytotoxicity, human patella-derived osteoblastic cell line, apoptosis and necrosis analysis

## Abstract

Regenerative medicine based on Mg alloy implants is considered a modern approach to address bone defects. It represents a promising alternative to traditional grafting strategies (auto-, allo-, and xenografts) by potentially mitigating complications such as donor-site morbidity and limited supply, which are discussed in this paper. In line with this global topic, attention is devoted to an innovative manufacturing route for Mg-Nd and Mg-Zn implants for the treatment of small bone defects. First, the proposed manufacturing method is described in detail, including the materials used and the manufacturing steps, and then a comparison between the reference (cast alloys) and implant samples is performed. The mechanical properties, weight loss in simulated body fluid (SBF), surface analysis (contact angle and roughness measurements), and cytotoxicity were evaluated to determine whether the developed implants are suitable for consideration as future bone implants. The main conclusions of the study were that both Mg-based implants exhibited mechanical properties (compressive strength and Young’s modulus) with values very close to those of the human bone, reduced mass loss (a fact that is in a direct relationship with an increase in corrosion resistance due to MgF_2_ conversion coating, which is a secondary result of the proposed manufacturing route), and finally, a good biocompatibility sustained by cell culture and cytotoxicity assessment, as well as by apoptosis and necrosis analysis on a human patella-derived osteoblastic cell line.

## 1. Introduction

The field of bone tissue engineering (BTE) has undergone rapid development, integrating biology, materials engineering, and medical knowledge to achieve practical applications. The primary goal of BTE is the complete or partial regeneration of bone defects. To achieve this, the structure must mimic the chemical composition and geometry of the extracellular matrix, ensuring the circulation of oxygen and nutrients. The biomaterials used must possess specific properties, including mechanical strength and elasticity similar to those of human bone, high biocompatibility, and, in some cases, biodegradability with a controlled degradation rate for optimal bone tissue healing and regeneration [1,2,3].

The design and practical implementation of three-dimensional (3D) implants play a crucial role in the framework of regenerative medicine, addressing organ malfunctions or soft or hard tissue loss. The advantages of using such types of implants are that they permit the regeneration of a high amount of the damaged natural tissue by offering adequate support for cell multiplication and differentiation [4,5]. It is well known that in BTE, the gold standard is characterized by autografts collected through dedicated methods from various parts of the human body. Unfortunately, this approach is not entirely safe due to an increased risk of infection, donor site morbidity, paresthesia, hematoma, or chronic inflammation. All of these side effects make a significant contribution to a prolonged hospitalization process [6]. Another solution that is encountered in practice is characterized by allografts obtained from other human donors; however, this procedure also poses major risks, such as graft rejection or disease transmission [6,7]. In certain cases, animal-origin xenografts may be involved. This treatment is associated with high drawbacks, such as bacteria or viruses’ transmission from animal to humans with unexpected adaptation of the pathogens and difficult cure, immunogenicity effects, and toxic host reaction associated with the sterilization procedure applied to the xenografts, which may, under certain circumstances, induce even graft rejection and threaten the patient’s life. It can be observed that artificial graft development is important because the side effects mentioned above do not characterize it, and implants are typically made from biomaterials that have already received approval for use on human subjects from different international organizations.

Today, there is no universally accepted definition of an ideal implant used in bone defect reconstruction with regenerative properties. Many researchers in the field emphasize that the implant must have a porosity higher than 50% in conjunction with a pore size between 200 μm and 300 μm. These conditions were obtained after many experimental analyses and investigations were performed, and the results were associated with increased cell adhesion and differentiation, as well as with adequate oxygen and nutritive substances circulation, concomitantly offering the possibility to eliminate the cell waste [8,9,10]. It was noticed that the chemical composition and the implant surface topography represent two important aspects that must be considered when cell adhesion and proliferation are discussed because the implant surface represents the main boundary between living tissue and biomaterial [11,12]. It is well known that surface roughness and umectability are two main factors contributing to osteoblast adhesion and differentiation. In addition, studies have shown that the implant elasticity module can guide mesenchymal stem cell differentiation towards osteoblasts and prevent the stress shielding effect from occurring if its values are close to those of human bone [13]. Generally speaking, the implant should support new bone formation both inside and outside its geometry. In the case of bioactive materials, this can be achieved through metallic ions or by-product emission, which can enhance the bone remodeling process. In conclusion, the pore distribution and shape, the porosity grade and pore interconnectivity, the implant penetration rate with cells, and the exposed surface can improve the osteogenesis process to a high degree [14].

One of the main challenges of BTE is the appearance of new blood vessels and the blood supply at the site of the bone defect, which play a vital role in bone viability insurance [15,16]. Another critical process that must be considered is osteoinduction, which represents the differentiation of stem cells into osteoblasts under the influence of the neighboring environment. This phenomenon is deemed essential to the bone regeneration process because it can directly influence treatment success. Implant osteointegration is described in the literature as forming a non-fibrous capsule, which acts as an interface between the implant and bone [17,18,19]. This way, the appearance of zones with osteoblasts is enhanced, allowing for new bone formation to be facilitated [20].

Typically, implants can be either permanent or temporary. For the first type, autoimmune reactions could appear, or a supplementary bone defect can be created as a result of the implant insertion step. Additionally, an increased concentration of metallic ions can be detected in the patient’s blood. Also, foreign body reactions, characterized by fibrous capsules that isolate the implant from human tissue and absorb non-specific proteins from the implant surface, can be observed. The permanent implants are made of titanium or its alloys, stainless steel, and Co-Cr alloys. In a study published in [21], the fact that titanium can induce allergic responses or even implant rejection was highlighted. Stainless-steel stress shielding and metallic ion emission have been reported, while premature implant failures have been observed in the case of Co-Cr alloys [22]. For all the materials mentioned above, studies presented in the literature underline the toxicity and secondary fracture appearance, with adverse effects on the initial bone defect healing process. They are inert materials that cannot establish a direct link with healthy tissue. Their primary option, as stated before, is to form a fibrous capsule around the implant. Bioactive materials that facilitate the artificial graft link to the host tissue were recently designed. This class includes ceramic materials such as hydroxyapatite, tricalcium phosphate, and bio-glasses [23,24,25]. Unfortunately, these materials do not exhibit good mechanical properties, are very fragile, and can be used as implant materials only for grafts dedicated to non-load-bearing zones. Other materials used in BTE are polymeric ones, including biocompatible and bioresorbable classes that have already been approved for use on humans. Drawbacks, such as by-product toxicity resulting from chemical reactions between materials and body fluids, reduced mechanical properties compared to those of human bone, and difficulty in processability, have been reported in the literature [26,27,28]. One of the most modern solutions adopted today is the use of porous magnesium (Mg)-based implants, which exhibit mechanical properties with values almost identical to those of trabecular or cortical bone [29,30,31]. It is well known that Mg rapidly degrades inside the human body due to chemical reactions with chloride ions, but its by-products do not exhibit a pronounced toxic character [19,32,33]. Additionally, it is notable that in the case of Mg alloys or coated implants, the corrosion rate can be significantly reduced, allowing for hydrogen emission to be considered within the biological range [2,34]. The Mg^2+^ ions have a beneficial effect on angiogenesis, osteogenesis, and the modulation of the anti-inflammatory state of macrophage cells, thereby positively influencing bone tissue regeneration and the treatment of chronic osteonecrosis.

In this study, a geometrical configuration of an experimental implant was designed and manufactured from Mg-Nd and Mg-Zn alloys, respectively, using an additive manufacturing procedure that combined a polymeric matrix with a titanium-based wire orthogonal weaving method. Based on the fused deposition technology, the geometry was printed in biodegradable and biocompatible polymeric materials. Then, through matrix pores, Ti6Al4V (medical Ti Grade 5) wires were introduced and woven. The polymeric matrix was dissolved in a dedicated solvent since the Ti-weaving structure was introduced in liquid Mg-based alloys, and Ti-Mg hybrids were obtained. After that, the Ti wires were removed using a hydrofluoric acid (HF) solution.

**Figure 1:**
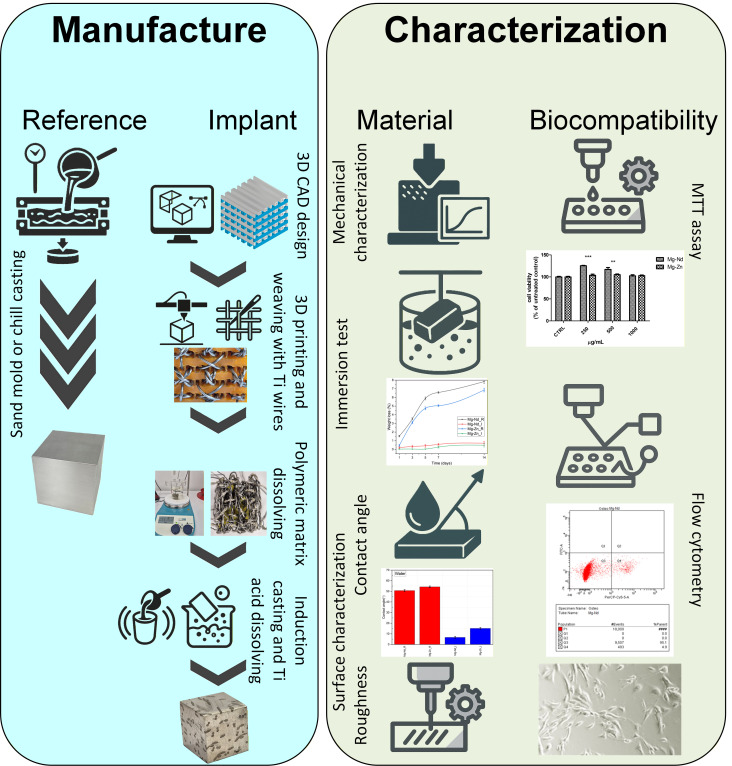
The main manufacturing steps and experimental characterization flowchart of the experimental samples.

Different types of samples (implant and reference) were mechanically characterized to establish whether their mechanical properties suit those of natural bone. Immersion analysis was performed to measure the weight loss at different time intervals in simulated body fluid (SBF). Then, the surface of the designed biodegradable implants in comparison with reference samples was analyzed based on contact angle measurements and profilometry. Cytotoxicity was assessed using extracts prepared from samples of implants on a human patella-derived osteoblastic cell line, while apoptosis and necrosis were analyzed based on the flow cytometry method. Figure 1 presents a flowchart of the manufacturing step and the characterization program for the experimental samples.

## 2. Materials and Methods

### 2.1. Implant Manufacturing Process

Two Mg-based alloys with the nominal chemical compositions presented in Table 1, denoted as Mg-Nd and Mg-Zn, were used to manufacture implants designed for healing minor bone defects through an innovative method, which will be explained and detailed later in the paper. The Mg-Nd alloy was obtained using a sand mold and chill casting method, while the second Mg-based alloy was obtained in the laboratory through a stir casting procedure. In the case of both alloys, the raw materials used were Mg (high purity, 99.99%), Y (high purity, 99.99%), Nd (high purity, 99.99%), Zn (high purity, 99.99%), Ca (high purity, 99.99%), and Mn (high purity, 99.99%), all sourced from Merck, Darmstadt, Germany. To validate the experimental composition, scanning electron microscopy (SEM) coupled with energy dispersive spectroscopy (EDS) was performed on these specific alloys in a previous study [35]. The EDS analysis revealed slight deviations from the nominal values (e.g., Mg-Nd contained approximatively 2.58% Nd, 0.38% Zn, and 0.32% Y; Mg-Zn contained approximatively 1.20% Zn, 0.54% Mn, and 0.35% Ca), which are attributed to the elemental recovery rates during the high-temperature melting and stirring processes. These deviations were found to be within acceptable limits and did not significantly alter the intended material properties.

The Mg-Nd and Mg-Zn implant and reference samples were synthesized based on high-purity chemical elements to minimize the presence of heavy metal impurities such as arsenic (As), mercury (Hg), lead (Pb), and cadmium (Cd). These high-quality starting powders ensure that all the impurities are at the ppm or ppb level. This affirmation is in line with the worldwide requirements for experimental biodegradable metallic implants [36].

The polylactic acid (PLA) was bought as a 3D printer filament with a wire diameter of 1.75 mm from Prusa Research, Prague, Czech Republic. This polymer is highly biocompatible and bioresorbable, and it is considered safe for implants dedicated to BTE [37,38,39].

The matrices used for the Ti alloy weaving procedure were prepared from this polymeric material. The wires from Ti6Al4V (Medical Grade 5) were bought from Stanford Advanced Materials, Lake Forest, CA 92630, USA.

The polymeric matrix was dissolved in a dedicated organic compound, as will be explained. Hydrofluoric acid (HF), with a concentration of 45% provided by Sigma-Aldrich, Saint Louis, MO, USA, was used to remove the Ti alloy wires and to generate a channel network within the implant.

In the first stage of the implant manufacturing process, a geometry for the PLA matrix was designed based on Autodesk Inventor^®^ Professional 2021. After that, it was 3D printed using the Fused Deposition Modeling (FDM) method with a Prusa i3 MK3S+ printer (Prusa Research, Prague, Czech Republic) and an extruder with a 0.2 mm diameter. Starting from the geometrical model, a file with the *.stl extension was prepared and imported into the PrusaSlicer 2.7.4 software for moving and repositioning it in the proper printing condition to locate its mass center at a decreased level. The printing speed was set at 0.2 mm/s, and the fill coefficient of the model was chosen to be 5%. Due to the robustness of the model, the polymeric matrix does not need a support structure.

The second stage of implant manufacturing consisted of using Ti6Al4V alloy wires with a diameter of 0.5 mm. These were inserted and woven into the polymeric matrix channels in a pattern that provided minimal mechanical support between Ti alloy wires to hinder deformation of the metallic structure after the polymeric matrix was dissolved. The Ti6Al4V-PLA hybrids were subjected to a chemical dissolution process to isolate the metallic structure. The samples were immersed in acetonitrile (AppliChem, Darmstadt, Germany) at a controlled temperature of 53–55 °C with a stirring speed of 800 rpm. Complete dissolution of the PLA matrix was achieved within 30 min. Post-dissolution, the remaining Ti6Al4V structures were ultrasonically cleaned in ethanol and dried.

After that, 3D-woven titanium wire structures were introduced together with small pieces of Mg-based alloys in an induction oven, resulting in a composite metallic material. The induction oven used in our study permits metal melting in an inert argon atmosphere and was manufactured by AAGES S.A., Sangeorgiu de Mures, Romania, with a nominal active power of 15 kW, a maximum voltage of 500 V, and a nominal apparent power of 20 kVA. To melt the two Mg-based alloys and generate the Mg-Ti6Al4V hybrid, the parameters for the magnetic field (mf) generator for the induction oven were set as follows: voltage V_mf_ of 363V, current I_mf_ of 9 A, and working frequency f_mf_ of 30 kHz.

Finally, the metallic composite samples were introduced in Berzelius glasses with a 45% concentration of hydrofluoric acid (HF), in an ultrasonic bath, to remove the Ti6Al4V wires from the metallic hybrid structures. The rationale of choosing 45% HF concentration was based on the fact that it generates a complete and fast dissolution of the Ti6Al4V wires concomitantly with a MgF_2_ conversion coating formation. At lower HF concentrations, the reaction between magnesium and water predominates, leading to the formation of magnesium hydroxide and oxide layers that yield thinner, less compact coatings. In contrast, higher HF concentrations favor the thermodynamically driven conversion of any Mg(OH)_2_ formed into MgF_2_, yielding dense and adherent protective layers. Drynda et al. [40] and Thomann et al. [41] proved that higher concentrations above 40% of HF led to a uniform and compact MgF_2_ layer formation compared to lower concentrations, and also this protective coating limited the H_2_ gas evolution in bone engineering applications to a great extent. Lower concentrations below 10% were linked to a reduced Ti-alloy wire dissolution speed and to a thinner conversion coating, while medium concentrations between 20% and 30% required longer immersion time for Ti-wire dissolutions [42].

### 2.2. Material Properties Evaluation

All the experimental results obtained on implant samples were compared with those for the reference samples made of the same cast Mg-based alloys. The coding of the implant and reference samples is presented in Table 2.

#### 2.2.1. Mechanical Properties

A compression test was carried out using a Walter + Bai LFV 300 system (Walter + Bai AG, Löhningen, Switzerland) and the ASTM E9-09 standard [43] was applied. The test was conducted under displacement-controlled mode at a constant speed of 5 mm/min. The measurement ended when the applied force dropped by approximately 75% from the peak load. The resulting stress–strain curves were recorded as the primary outcome.

#### 2.2.2. Immersion Test in Simulated Body Fluid

The corrosion process was investigated based on immersion tests. It was chosen simulated body fluid (SBF) because it has a chemical composition similar to that of human blood plasma [44,45,46]. The SBF was prepared in the laboratory using the Kokubo protocol [47] and characterized by a physiological pH of 7.4. The medium was changed daily, and a temperature of approximately 37 ± 5 °C was maintained using a thermostatic bath (Julabo Water Bath, Julabo GmbH, Seelbach, Germany). Tests were conducted in 280 mL of SBF, in accordance with the ASTM G31-21 standard, which recommends a solution-to-specimen ratio of 20 mL/cm^2^ or 40 mL/cm^2^ [48]. To standardize the degradation behavior, the implant and reference samples were prepared with a total computed surface of 6.99 cm^2^, leading to an SBF-to-sample ratio of 40 mL/cm^2^. This volume was selected to provide a sufficient buffer against the pH increases due to magnesium degradation and to ensure that the results accurately reflect the different degradation behaviour of reference and implant samples.

The sample weight was measured at different intervals (1, 3, 5, 7, and 14 days) in triplicate before and after the soaking procedure. Corrosion behavior was investigated by computing the sample weight loss, as presented in [33,49] based on Equation (1).

(1) $$w_{L}(\%)=\frac{w_{i}-w_{f}}{w_{i}}\cdot 100,$$

where *w_i_* (g) is the initial weight, while *w_f_* (g) is the weight at the end of the process.

The rationale for choosing a 14-day immersion time was based on the observation that, usually, in the case of Mg-based alloys, the first two weeks are characterized by an important corrosion effect, being considered as a critical kinetic window time. In [50,51,52], it is stated that the most aggressive degradation process occurs between the first 24 to 72 h, because H_2_ emission and rapid weight loss happen during the initial damage of the surface oxide layer. At 14 days, a quasi-passive layer is formed, making this time period significant for a long-term degradation investigation. Increasing the test duration over 14 days could damage the implant’s structural integrity, making weight loss measurements inaccurate. In addition, in BTE, the 14-day period is critical for cell proliferation and attachment, leading to the soft callus formation, due to the fact that toxic pH values of the implant environment or gas pocket occur in the first 14 days. A study of Mg-Zn-Ca reported an initial degradation rate of 1.5 mm/year over the first 7 days, followed by stabilization at 0.3–0.5 mm/year between 14 and 60 days [53]. Also for Mg-Nd, a steady degradation rate of about 0.12 mm/year was observed from day 30 to day 180 [54], so as a direct consequence, 14 days is a correct baseline for long-term degradation behavior assessment.

#### 2.2.3. Surface Properties

DSA 100 Krüss Drop Shape Analyzer (A. Krüss Optronic GmbH, Hamburg, Germany) was used to investigate the sample wettability. This device permits contact angle measurements based on three liquids: water (W), ethylene glycol (EG), and diiodomethane (DIM). All the measurements were made in a controlled medium characterized by 45 ± 5% humidity and a temperature of 23 ± 5 °C. The contact angle was determined for 5 samples for each type of sample category.

Then, Owens, Wendt, Rabel, and Kaelbe’s (OWKR) method, dedicated to separating the interfacial tension into two components that characterize the so-called polar and dispersive interactions, was applied for the computation of surface free energy (SFE) [55]. Keesom forces are directly linked to the polar interactions [56], while London forces [57] are known in the literature as dispersive components.

Sample profilometry measurements were performed based on a Form Talysurf^®^ I-Series PRO Range Taylor Hobson Ametek (Warrenville, IL, USA) equipment. This device can analyze surface roughness according to the ISO 21920 standard [58] and is compatible with Metrology 4.0 Software (Taylor Hobson, Ametek, IL, USA). It is based on a quartz spheroidal standard probe and a transducer.

The roughness measurements consisted of five consecutively executed observations, and the arithmetic average deviation from the mean line (R_a_) and root mean square average of the profile heights over evaluation length (R_q_) values were provided.

### 2.3. In Vitro Biocompatibility Analysis

#### 2.3.1. Cell Culture and Cytotoxicity Assessment

Human patella-derived osteoblastic cells [59] were thawed, cultured to approximately 80% confluence, and enumerated using a benchtop-sized counter, EVE^TM^ Automatic Cell Counter (NanoEntek, Seoul, Korea). Cell viability was assessed using the Trypan Blue exclusion assay. The cells were seeded into 24-well plates at a concentration of 1 × 10^5^ cells per well and maintained in Dulbecco’s Modified Eagle Medium/Nutrient Mixture F-12 (DMEM/F-12) medium supplemented with 15% fetal bovine serum, 1% Glutamax, 1% non-essential amino acids, and 1% antibiotic–antimycotic solution. After 24 h under standard incubation conditions (37 °C, 90% relative humidity, 5% CO_2_), the medium was replaced with fresh medium containing three different dilutions of 250, 500, and 1000 μg/mL, respectively, conditioned media derived from both Mg-Nd_I and Mg-Zn_I. The conditioned media were generated 48 h prior to treatment by incubating the implant extract samples in culture medium, followed by filtration before use. Each experimental condition was tested in triplicate, and untreated cell cultures served as negative controls. Cytotoxicity was determined 24 h post-treatment using the Cell Counting Kit-8 assay (CCK-8) following the manufacturer’s guidelines. Absorbance at 450 nm was measured with a BioTek Synergy^TM^ 2 microplate reader (Agilent, Bucharest, Romania), and cell viability was expressed as a percentage relative to the untreated control samples. The rationale for choosing the extract concentrations between 250 ug/mL and 1000 ug/mL was based on the fact that these concentrations represent a burst release of Mg^2+^ ions observed during the initial stages of biodegradation and the values were established in a direct relationship with bibliographical studies and preliminary toxicity tests [60]. In addition, the upper limit of 1000 ug/mL is a standard value used in many Mg-based alloys or implants for cytotoxicity studies to determine the metabolic stress in fibroblasts and osteoblasts [61,62,63]. Also, the ISO 10993-5 standard states that testing of high concentrations permits the establishment of a dose–response curve that ensures that even if a localized fast degradation occurs, the ion concentration remains within the cytocompatibility limits [64].

#### 2.3.2. Apoptosis and Necrosis Analysis

Apoptotic and necrotic cell death were assessed after exposure to the Mg-based implant extracts using an Annexin V/Propidium Iodide (PI) staining assay (Cat. 30017, Lot 210723). Following 24 h of treatment with inserts containing Mg-Nd_I and Mg-Zn_I sample at 1000 μg/mL, cells were collected, labeled with Annexin V and PI, and analyzed using a BD FACSCanto^TM^ II flow cytometer (Becton Dickinson (BD), Franklin Lakes, NJ, USA) equipped with 488 nm argon and 633 nm helium–neon lasers. Unstained samples were used to define gating settings and eliminate debris. Fluorescence signals were detected with a 525/50 bandpass filter for Annexin V and a 695/40 filter for PI, and 10,000 events were recorded per sample at the lowest flow rate. Based on quadrant analysis, cells were categorized as viable (Annexin V^−^/PI^−^, Q3), early apoptotic (Annexin V^+^/PI^−^, Q1), late apoptotic (Annexin V^+^/PI^+^, Q2), or necrotic (Annexin V^−^/PI^+^, Q4). Data were reported as percentages of the total cell population.

#### 2.3.3. Statistical Analysis

All data from the cell viability assays are presented as the average value (mean) ± standard deviation (SD). Before, data distribution was assessed for normality using the Shapiro–Wilk test. As no significant deviations from normality were observed, statistical analysis was performed using two-way analysis of variance (ANOVA), followed by the Bonferroni post hoc test. The threshold for statistical significance was set at *p* < 0.05. Where applicable, higher levels of significance (*p* < 0.01 and *p* < 0.001) are indicated in the text and figures to denote the degree of statistical confidence. The cytotoxicity assay studies were performed in three independent biological replicates, each conducted with technical triplicates (*n* = 3), in line with common practice for exploratory in vitro cytocompatibility screening. Sensitivity power analysis indicated that the study was powered to detect large effects. Statistical analyses and graphical representations were generated using GraphPad Prism software (Version 8.0; GraphPad Software, San Diego, CA, USA). The rationale for choosing a sample size of *n* = 3 is considered an equilibrium between experimental feasibility and statistical validity. To perform a very accurate analysis, the two-way ANOVA was combined with the Bonferroni post hoc test. The last-mentioned analysis significantly reduces the risk of false-positive errors, which is common in multiple comparisons. By considering the high magnitude of difference between the control and experimental group, this sample size was sufficient (*p* < 0.05). In addition, the narrow SD bars in the figures sustain the sample size.

## 3. Results and Discussion

### 3.1. Implant Design Outcomes

**Figure 2:**
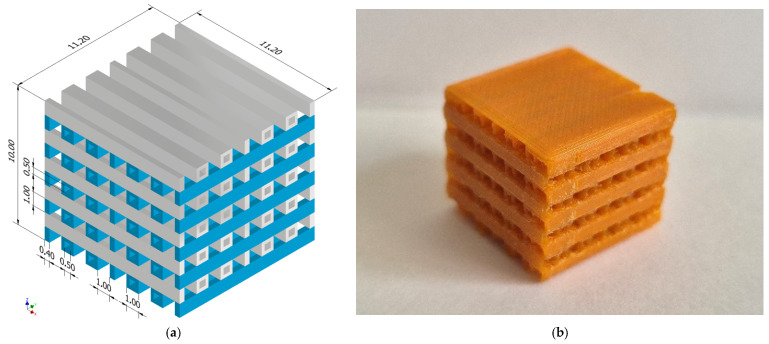
Polymeric matrix and Ti6Al4V-PLA hybrid used in the first stage production process of the Mg-based implants. (**a**) The CAD geometry of the PLA matrices [65] (Figure is licensed under CC-BY 4.0); (**b**) practical implementation of the PLA matrix.

Figure 2 [65] presents the geometrical configuration and practical implementation of the PLA matrix, as described in Section 2.2.

It can be observed that the printed structure accurately respects the geometrical dimensions of the initial model [65]. In addition, there were no deformed printing layers or rounded corners, which are considered fabrication defects. Typically, nozzle size, filament diameter, printing speed, layer height, and fill density are key parameters that can impact the quality of the printed samples. The nozzle size determines the height of the printed layers, while the printing speed should be considered in relation to the polymer extrusion rate to achieve a uniform and continuous printing wire. In this study, the extruder diameter was chosen to be equal to 0.2 mm, which provides the PLA matrix with good interlayer bonding. The printing parameters of the Prusa i3 MK3S+ are provided in Table 3.

The rationale for selecting the printing parameters presented In Table 3 Is based on the fact that by choosing a 0.2 mm extruder and a very slow printing speed of 0.2 mm/s, a strong condition for high-fidelity reproduction was achieved. For instance, in the case of an implant with an 11.2 mm edge of the base, the nozzle-to-part ratio is about 1:56. This permits the gyroid architecture manufacture with a wall thickness corresponding to a single or double ‘bead’ width, ensuring that the trigonometric curvature of the gyroid was captured without the ‘staircase’ approximations common in larger-nozzle systems. In addition, to ensure the fidelity of the gyroid’s non-linear paths, an ultra-low printing speed of 0.2 mm/s was used. This near-quasi-static deposition rate determines the laminar flow of the polymer and allows for instantaneous cooling and solidification of the gyroid struts. This fact minimizes thermal shrinkage and geometric warping, which are the primary causes of non-reproducibility in small-scale porous implants. At a 5% fill density, the gyroid unit cell is characterized by relatively large open pores and thin structural walls. Given the computed total volume of the implant, the calculated wall thickness of the gyroid template remains significantly above the 0.2 mm/s resolution limit of the extruder. This ensures that the pore interconnectivity is respected over all implant height. Additionally, the visual inspection of the G-code proved that the gyroid pattern was successfully resolved into continuous, non-intersecting paths. The 60 °C bed temperature provided sufficient adhesion to prevent ‘base-shift,’ which is critical when printing high-aspect-ratio features like the thin walls of a 5% density gyroid.

During the second step of the production process, medical Ti6Al4V alloy wires were woven as previously presented. Additionally, the PLA material was correctly chosen for the 3D printing process of the initial matrix, as the weaving procedure was easy to fulfill and the polymeric structure remained intact, with good adhesion properties between the printed layers.

**Figure 3:**
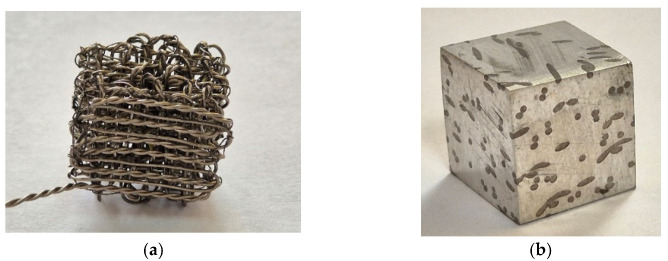
Ti6Al4V structure and Mg-Ti6Al4V hybrid. (**a**) Ti6Al4V wire weaving structure; (**b**) Parallelepipedal Mg-Ti6Al4V alloy hybrids.

The Ti6Al4V-PLA alloy hybrid was inserted into a dedicated solvent to dissolve the polymeric matrix (Figure 3a).

In Figure 3b, an example of a hybrid metallic sample prepared based on Mg-Nd or Mg-Zn alloys is presented.

**Figure 4:**
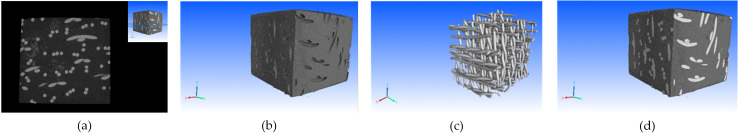
Investigation images of the Mg-Ti6Al4V hybrid: (**a**) CT-2D cross-sectional view of the hybrid; (**b**) CT-3D view of the Mg-based alloy structure; (**c**) CT-3D view of the Ti6Al4V wire weaving; (**d**) 3D view of the Mg-Ti6Al4V hybrid.

Computed tomography was used to highlight the Mg-Ti6Al4V hybrid structure. It involved a CT Scanning Device (XT H 225 model, Nikon Corporation, Tokyo, Japan). After the scanning process, the obtained images were investigated with VGSTUDIO MAX Program (2024.3 software version) (Figure 4). By considering the geometrical dimensions of the samples, presented in Figure 2a, and the type of the CT device, the voxel size was 10 μm. Based on the classical Nyquist–Shannon sampling theorem and the limitations of “geometric unsharpness” in the X-ray tubes, the standard device resolution is about 2 to 3 voxels. For the specific investigated samples, the minimum detectable defect size is between 20 and 30 μm [66]. The performed CT analysis is in accordance with the CT metrology standards [67,68], in which the minimum detectable size should be 2–3 times higher than the voxel dimension, as stated also in the VGSTUDIO MAX (2024.3 version).

**Figure 5:**
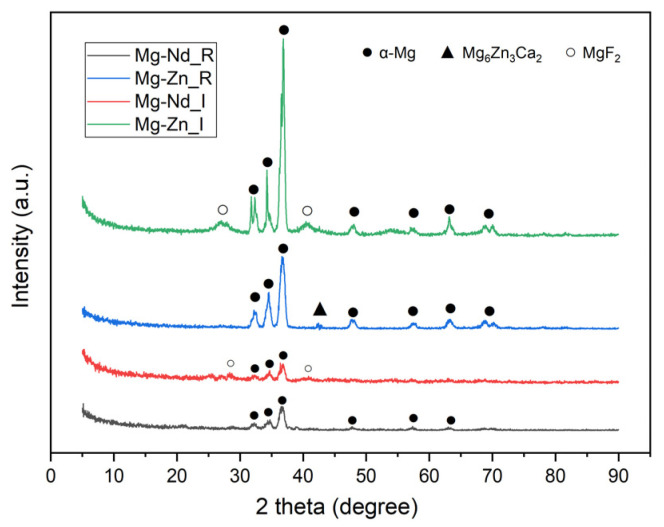
X-ray diffraction patterns of the implant and reference samples.

It can be concluded that the induction melting method was correctly chosen because the Ti6Al4V weaving structure was completely immersed in liquid Mg alloy. Based on this technology, hybrid Mg-Ti6Al4V alloy samples with an almost uniform morphology were obtained. After that, the Ti6Al4V wires were dissolved in a 45% concentration of HF, and the final implant was obtained. It is well known that a second result of the Mg immersion process in HF through a chemical conversion process could appear as a coating consisting mainly of MgF_2_, which is beneficial for corrosion rate decrease, exhibiting an increased biocompatibility with no implications on cell viability or side effects such as necrosis due to an excess hydrogen accumulation [69,70,71,72]. The alloy phase and fluoride conversion coating were evidenced based on a PANALYTICAL X-Pert PRO diffractometer (Malvern Panalytical, Worcestershire, UK) equipped with CuKα radiation. The measurement results were processed in PDXL Software 1.2.0.1 (ICDD 1999). The obtained results for the reference samples are in good agreement with those presented in [35], while in the case of both implant samples, an additional peak corresponding to MgF_2_ was identified as expected (Figure 5).

Just to summarize the mechanism of MgF_2_ formation as a consequence of immersion of Mg-Nd and Mg-Zn implant samples in an aqueous solution of HF, one must mention that this process is a topochemical conversion, which is conducted by the high chemical potential of 45% HF based on the following chemical reactions (Equations (2)–(8)) [73,74]. One can immediately notice that Mg^2+^, H_2_, and OH^−^ occur as a result of an intense corrosion process of the Mg-based alloys. Then Mg^2+^ reacts with F^−^ and OH^−^, and different compounds are formed (Equations (5)–(7)). Mg(OH)_2_ is unstable under acidic conditions and undergoes an exchange chemical reaction (Equation (8)), in which OH^−^ is replaced by F^−^.

(2) $$Mg\to Mg^{2+}+2e^{-},$$

(3) $$2H^{+}+2e^{-}\to H^{2}↑,$$

(4) $$2H_{2}O+2e^{-}\to 2OH^{-}+H_{2}↑,$$

(5) $$Mg^{2+}+2F^{-}\to MgF_{2},$$

(6) $$Mg^{2+}+2OH^{-}\to Mg{\left(OH\right)}_{2},$$

(7) $$Mg^{2+}+XF^{-}+(2-X)OH^{-}\to Mg{\left(OH\right)}_{2-x}F_{x},$$

(8) $$Mg{\left(OH\right)}_{2}+2F^{-}\to MgF_{2}+2OH^{-}.$$

At this acid concentration, a fast supersaturation effect is present at the interface between the metal and the liquid and generates a dense fluoride film [70,75].

In the direct relationship with the implant manufacturing route, some key process performance metrics should be mentioned. In regard to the reproducibility, it is directly linked to the Ti6Al4V wire diameter and the manual weaving procedure. The method developed in the paper could be considered similar to the titanium wire space holder one. In Cheng et al. [51] and Antoniac et al. [29] it was proved that in most cases the pore size is almost the same as the Ti alloy wire diameter, with reduced deviations of about ±(2 – 5)%. In addition, the polymer template is used only as a secondary structure that permits the weaving procedure of the Ti6Al4V wires. Then the Ti structure remains intact during the liquid Mg-based alloy infiltration process.

As stated in the literature, Ti-wire-templated Mg alloy implants exhibited a total porosity within 1.5% of the designed model [76]. Additionally, due to the fact that the wires are manually woven, a channeled structure is obtained without dead-end pores, an important advantage compared to powder metallurgy. In [65], a porosity was determined based on a mathematical formulation.

It Is worth mentioning that, as already explained, the use of hydrofluoric acid not only removes the Ti6Al4V wires but also generates a protective and biocompatible conversion coating of MgF_2_. Schmidt et al. [77] showed that the surface atomic ratio of F:Mg is about 2:1, a fact that increases the corrosion resistance of the implant. This post-processing step is useful to clean the implant channel from polymeric residues concomitantly with surface passivation.

Cyclic testing will be a future task, but the structural integrity of the weave implant geometry was already validated in a previous work [65]. The model that was chosen to be practically implemented demonstrated that under physiological loading, the stress distribution is uniform. Also, based on the fatigue life predictions met in the literature performed on Mg implants with 40–55% porosity, the failure cycles are estimated to be about 3–4 months [78,79].

### 3.2. Material and Implant Properties

#### 3.2.1. Mechanical Characterization

According to [35], the production method for Mg-based alloys is of utmost importance when mechanical properties are analyzed. As stated previously, in the case of cast alloys compared to those obtained through different procedures, the coarse-grain structure results in lower values of compressive strength and Young’s modulus [80,81,82].

**Figure 6:**
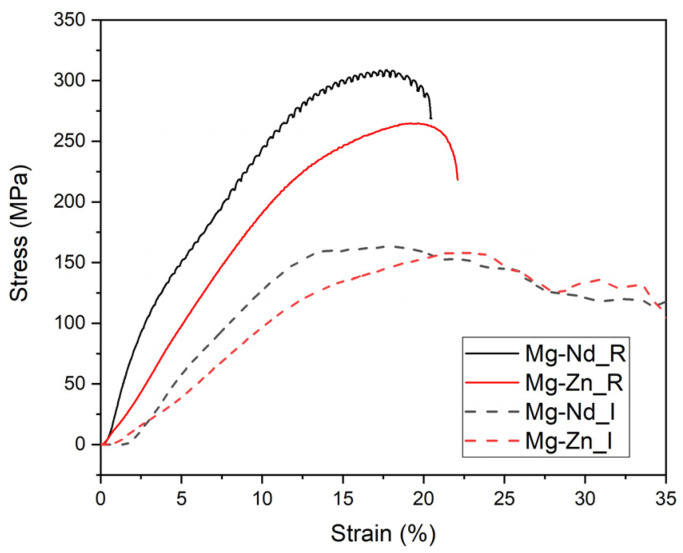
Stress and strain graphs obtained after the compression test of reference and implant samples manufactured from Mg-Nd and Mg-Zn alloys.

The compression analysis was conducted on five samples prepared from cast (reference) or implant materials, respectively, in accordance with the ASTM E9-09 standard [43]. Figure 6 shows the stress–strain variations obtained for the implant and reference samples. One can immediately notice that the Mg-Nd reference and implant samples exhibited a stronger strain hardening phenomenon. For Mg-Zn probes, a higher deformation capacity was evidenced.

Regarding the compressive strength, we obtained higher values for the reference samples (Mg-Nd_R: 320.210 ± 13.400 MPa, Mg-Zn_R: 289.12 ± 12.150 MPa) compared to the implant samples (Mg-Nd_I: 187.550 ± 11.301 MPa, Mg-Zn_I: 158.210 ± 11.500 MPa). It can be concluded that the implant samples are characterized by compressive strength values that are much closer to those of human bone (205 MPa along the longitudinal axis and 131 MPa in the transverse axis [83]), demonstrating that the proposed design is suitable for use as a bone substitute. In case of the Young’s modulus, when compared to the value of natural bone (trabecular bone 14.800 ± 1.40 GPa, cortical bone 20.700 ± 1.90 GPa) [83], the implant samples had similar values (Mg-Nd_I: 16.79 ± 0.50 GPa, Mg-Zn_I: 10.27 ± 0.7 MPa). For reference samples, we have provided values in [35]. Their Young’s module exhibited a slightly increased value, which can be associated with higher stiffness. This fact is accompanied by a reduction in the area under the stress–strain curve prior to failure, suggesting a more brittle fracture mode. This behavior can be attributed to the specific Mg-based alloy microstructure and the ‘weave’ geometry, which facilitates high load-bearing capacity but limits the capacity for plastic energy absorption before strut fracture. The implant samples exhibited a ductile character, which could be attributed to the presence of internal channels.

To evaluate the overall mechanical stability of the implants, a one-way ANOVA was performed on the integrated stress–strain data (Energy Absorption Capacity) for the independent replicates (*n* = 5). The analysis revealed a high statistical significance among the alloy groups (F = 18.42, *p* < 0.001). The results demonstrated that the mean values of the mechanical properties are indeed statistically significant, highlighting the reliability of our findings.

The obtained results are in good agreement with the literature, as presented also in [35]. The mechanical performance of magnesium-based systems varies significantly based on composition and processing history. For instance, the compressive strength of 337 ± 3 MPa reported for Mg-Al-Zn-Mn alloys under high strain rates (2900 s^−1^) aligns closely with the values observed in the current study [82]. In contrast, as-cast Mg-Fe-Ca alloy exhibits a much lower strength of 165.6 MPa, which can be enhanced to 209.7 MPa through thermal treatment (500 °C for 4 h) [84]. This improvement is typically credited to the dissolution of calcium into the magnesium lattice and the resulting refinement of the grain structure. Alloying strategies also play a decisive role; Zn additions have been shown to elevate compressive strength to approximately 250 MPa, a substantial increase over the 92–95 Mpa range typical of sintered pure magnesium [85]. Furthermore, the selection of ternary elements like Sr, Zr, or Sn in Mg-0.5Ca systems can shift compressive strengths between 150 and 350 MPa, while maintaining a Young’s modulus of roughly 45 GPa [86].

#### 3.2.2. Biodegradation Evaluation After Immersion in Simulated Body Fluid

One must mention that SBF represents an important medium for bioactivity analyses; however, for degradation behavior, it exhibits some limitations, as it mimics ionic plasmatic concentrations very well but lacks the attributes of a real biological environment [87]. Firstly, SBF does not contain albumin or other plasma proteins, which differs from in vivo tests because proteins usually adhere to Mg-based implant surfaces and reduce the initial corrosion spikes [88]. Another limitation is that SBF uses TRIS or CO_2_ to maintain pH, whereas living organisms have a complex bicarbonate-based buffering system [89]. It is expected that in a static SBF investigation, a rapid increase of pH could occur, and a faster corrosion process could develop when compared to in vivo conditions [90]. SBF immersion analyses are static, and the living models are characterized by biological fluid flow, and differences between results are noticed. Another limitation is the absence of cells in the fluid medium, so no biological remodeling is expected. On the other hand, SBF is a worldwide scientific medium, and the obtained results could be easily compared with the existing literature. SBF induces apatite formation, being superior to NaCl solution [91], and is unanimously considered a conservative medium for alloy degradation tests.

**Figure 7:**
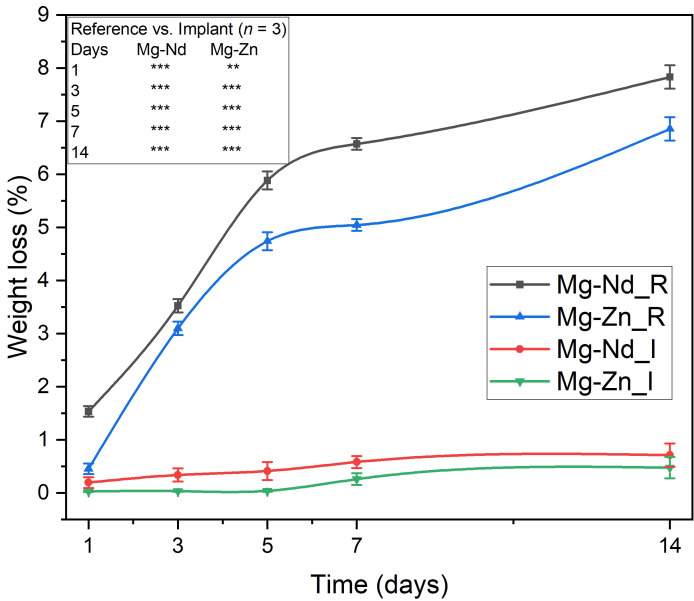
Weight loss (%) of reference and implant samples in the case of both tested alloys after 1, 3, 5, 7, and 14 days of immersion in SBF. Data are presented as mean ± SD (*n* = 3). Statistical significance between the reference and implant groups at each time point was determined via Student’s *t*-test (** *p* < 0.01; *** *p* < 0.001). The results indicate a highly significant reduction in weight loss for the implant samples at all time points (*p* < 0.001 for nearly all comparisons), confirming the protective efficacy of the MgF_2_ conversion coating.

The variation of the degradation rate Investigated based on weight loss computation is shown in Figure 7. It can be observed that for both investigated Mg-based alloys, the implant samples, which were immersed in HF during the manufacturing process, exhibit a reduction in degradation when introduced into SBF at human body temperature.

From Figure 7, it can be observed that the Mg-Zn_R sample degrades in a much slower manner compared to the Mg-Nd_R sample until day 3 of the experiment (Mg-Zn_R: day 1—0.455%, day 3—3.099%, day 14—6.855%). On the other hand, the Mg-Nd_R exhibits a mass loss proportional to the increase in immersion days, starting from 1.536% at day 1, 3.526% at day 3, and reaching 7.833% at day 14. As obtained from the electrochemical analysis described in [35], it can be observed that Mg-Zn_R and Mg-Nd_I samples exhibit reduced weight loss. In addition, for implant samples, very low values for mass loss were measured (day 14: Mg-Nd_I: 0.712 g and Mg-Zn_I: 0.475 g). It can be concluded that the fluoride-coating layer offered a protective action against corrosive agents, such as the SBF solution.

The obtained results are In good agreement with the literature [92]. In Ref. [93], an interesting analysis was developed regarding the effect of fluoride treatment on degradation in the case of Mg-Ca alloys. It was clearly shown that the surface treatment performed by soaking in a 35% concentration HF solution generated a lower mass loss value of approximately 0.015 g at 168 h of immersion in SBF solution, compared to 0.017 g for the 40% concentration HF solution and 0.038 g for the untreated sample. It can be concluded that the formation of the MgF_2_ protective layer results in increased corrosion resistance, and the loss of mass can be considered a function of the HF concentration. Rezaei-Baravati et al. [94] established that soaking time in HF of Mg-Zn-Al-0.5Ca is very important in the case of immersion tests. The lowest value of weight loss was approximately 0.015 g for the samples immersed in HF for 24 h, at 192 h of soaking in SBF. The weight loss gradually increased by decreasing the time of surface treatment, with 0.028 g obtained for 6 h HF-immersed samples at 192 h, and at about 0.037 g in the case of untreated alloys. It was concluded that the surface treatment time is of utmost importance in controlling weight loss and adapting the material interface to different applications.

#### 3.2.3. Surface Characterization

Surface wettability is crucial in determining whether an implant is beneficial for cell adhesion and proliferation, and whether it can provide a satisfactory biological response. In the case of hydrophilic surfaces, a high grade of implant osteointegration occurs due to an increased cell multiplication process that permits the formation of a link between living tissue and biomaterial. For hydrophobic surfaces with contact angles higher than 90°, a low rate of molecule and protein absorption was reported. It was concluded in many studies [95,96,97] that these types of surfaces are not ideal for implants, which must be fully integrated into the human body.

As previously stated, the OWKR method that consists of using three liquids (water (W), ethylene glycol (EG), diiodomethane (DIM)) was applied for contact angle (CA) measurements. In the case of water as a wetting agent, it can be observed that the reference Mg-Nd_R sample has a slightly decreased value of the contact angle of 50.695 ± 0.802° compared to the reference Mg-Zn_R probe with a value equal to 54.234 ± 0.788°. The same result was obtained also for diiodomethane fluid (Mg-Nd_R: 38.575 ± 0.800°, Mg-Zn_R: 39.819 ± 0.934°). In the case of the ethylene glycol wetting agent, the cast Mg-Nd_R had a higher value compared to that obtained for the other reference samples (Mg-Nd_R: 62.731 ± 0.875°, Mg-Zn_R: 56.865 ± 0.905°). It can be concluded that both alloys exhibited similar wettability behavior, a fact that can be attributed to their comparable chemical compositions.

**Figure 8:**
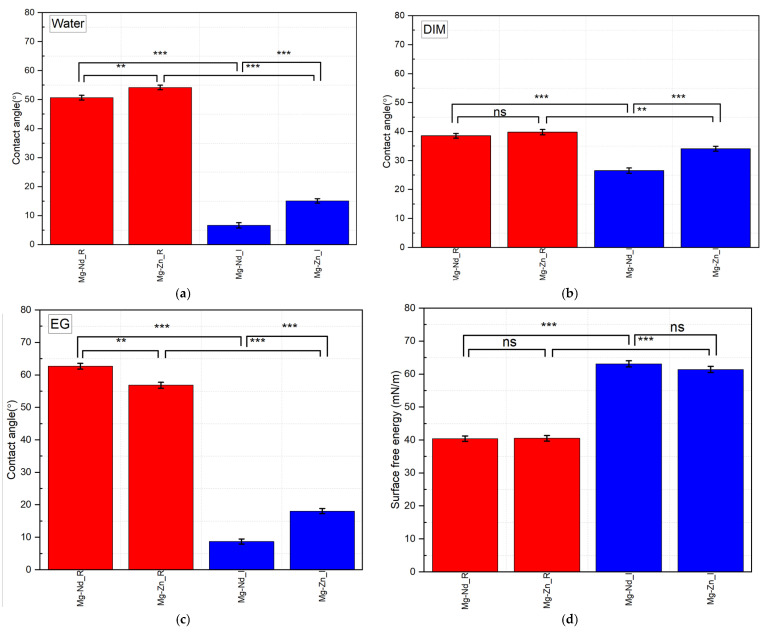
Contact angle values with their standard deviations in the case of the three wetting agents and surface free energy. (**a**) CA measured for water—the implants exhibited a highly significant (*p* < 0.001) reduction in contact angle, indicating a transition to a super-hydrophilic state; (**b**) CA measured for diiodomethane—the implants are characterized by an important reduction of the CA, confirming a major shift in the surface’s dispersive properties; (**c**) CA measured for ethylene glycol—implants have a highly significant (*p* < 0.001) reduction in the ethylene glycol contact angle for both Mg-Nd and Mg-Zn alloys, further supporting the comprehensive energetic characterization of the passivated surfaces; (**d**) SFE values computed based on the OWKR method—the MgF_2_ coating resulted in a highly significant (*p* < 0.001) increase in total surface free energy, providing a superior thermodynamic environment for protein adsorption. All the data are presented as mean ± SD (*n* = 5). Statistical significance was determined via Student’s *t*-test (ns *p* > 0.05; ** *p* < 0.01; *** *p* < 0.001).

On the other hand, the Mg-Nd_I sample had a more hydrophilic surface compared to the other investigated Mg-Zn_I in the case of the three wetting agents (Figure 8a–c). For example, for the Mg-Nd_I contact angle was equal to 6.653 ± 0.905°—W, 26.533 ± 0.913°—DIM, and 8.721 ± 0.788°—EG, while in the case of Mg-Zn_I the following values were obtained: 15.108 ± 18.086°—W, 34.114 ± 0.856°—DIM, and 18.086 ± 0.805°—EG.

The surface free energy (SFE) components were determined per the OWKR method [55], considering that diiodomethane is a nonpolar/dispersive liquid, and water and ethylene glycol are polar liquids. It is well known that the surface contact angle can be correlated with SFE values in an inverse-proportional fashion. A material surface with an adequate biocompatibility grade is characterized by a high value of SFE and a decreased CA. Still, the cell behavior can be correctly described after the analysis of wettability is investigated in relation to the surface chemical composition, roughness, and topography [98,99]. From Figure 8d, it can be noticed that the SFE increased after the conversion coating was achieved during the implant manufacturing stage, from 40.430 mN/m (Mg-Nd_R) to 63.137 mN/m (Mg-Nd_I) and from 40.540 mN/m (Mg-Zn_R) to 61.418 mN/m (Mg-Zn_I), respectively.

From the contact angle analyses, it can be concluded that the implant made of alloy Mg-Nd becomes more hydrophilic after the manufacturing process and that the fluoride conversion layer favors the osteogenesis process, which is beneficial to cell proliferation and adhesion.

The wettability measurements are in good agreement with the literature. Bita et al. [100] investigated the effect of fluoride coating on the corrosion behavior of Mg-Zn-Ca-Mn alloy dedicated to biomedical use. The authors obtained a contact angle value of 51.51 ± 0.76° for water as a wetting agent in the case of the pristine sample and a value of 14.94 ± 0.56° after the alloy immersion in HF. They concluded that the fluoride coating led to a highly hydrophilic surface. In a study by Quan et al. [101], a similar fluoride treatment was applied to enhance the corrosion resistance of Mg-Nd-Y-Zn-Zr alloys. A contact angle value of 55 ± 0.987° was obtained for the untreated alloy, while for the coated samples, a significantly more hydrophilic surface with a reduced contact angle of 23 ± 0.331° was observed. Ashong et al. [102] analyzed the effect of HF treatment on the mechanical properties and wettability of laser-bonded Mg-3Al-1Zn (AZ31) alloy. It was observed that the HF-treated samples exhibited a significantly lower contact angle of 8° compared to the control samples, which had a contact angle of 71°. The main conclusion of this study was that combining laser treatment with HF immersion can achieve super-hydrophilicity of the surface. Xu et al. [103] investigated the effect of various concentrations of HF treatments on the wettability of Mg-3Al-1Zn (AZ31) stents. It was found that 23% and 46% HF concentration-treated samples presented a reduction of the contact angle from 82.4° to about 10°. The authors concluded that the conversion coating resulted in a surface transformation from one that was almost hydrophobic to one with enhanced hydrophilic properties.

By comparing the contact angle values obtained for reference samples made of cast Mg-based alloys, it can be observed that the materials initially exhibited a similar wettability behavior, with average contact angles of around 55°. Still, after the coating layer formation during the implant manufacturing stage, the contact angles decrease by a high amount for both samples. For example, the Mg-Nd_I sample exhibited the lowest value of 6.653 ± 0.905° compared to the 15.108 ± 18.086° obtained for Mg-Zn_I, which makes both implants suitable for use in orthopedics to alleviate bone defects due to their hydrophilic surface wettability.

Surface roughness is important in the material analysis, and a direct link can be established with cell proliferation and adhesion [104,105], osteogenesis, and implant osteointegration [106,107], and the surface degradation process [108,109,110]. The influence of the roughness on wettability was investigated in different studies based on the so-called Wenzel roughness factor [111,112], included in a model that presented that the roughening surface process could generate physicochemical modifications that can change the wettability and surface free energy. This theory states that an increase in alloy surface roughness generates a much more pronounced hydrophilic character if, before this surface change, the material presents a contact angle value below 90°.

**Figure 9:**
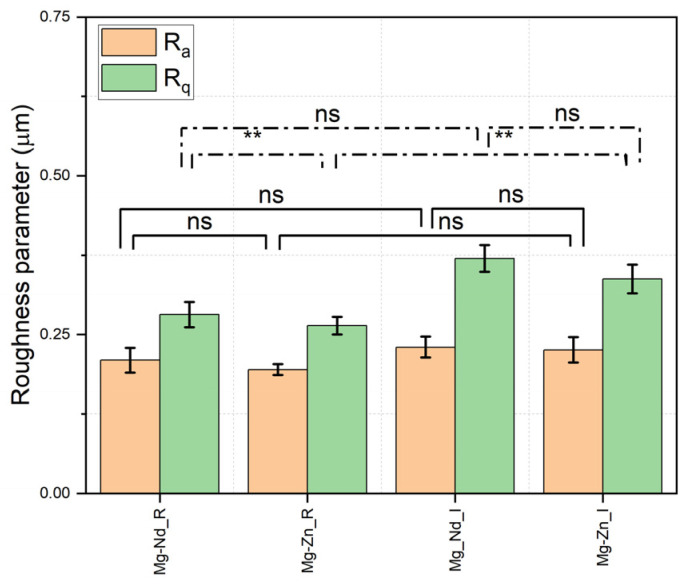
Roughness parameter (R_a_ and R_q_) values obtained in the case of reference samples and implant samples: R_a_ (solid brackets)—the statistical analysis confirms that the conversion coating does not significantly alter the average roughness of the implants, and it suggests the protective MgF_2_ layer is thin and conformal, preserving the designed surface micro-topography; R_q_ (dash dot brackets)—the analysis reveals that the passivation process significantly modifies the root mean square roughness of both Mg-based implants (*p* < 0.01). All the data are presented as mean ± SD (*n* = 5). Statistical significance was determined via Student’s *t*-test (ns *p* > 0.05; ** *p* < 0.01 ).

The roughness parameters determined based on profilometry analysis exhibited increased values in the case of implant samples compared to the reference probes for both Mg-based alloys (Figure 9) (e.g., Mg-Nd_R: R_a_ = 0.209 ± 0.019 μm, R_q_ = 0.281 ± 0.019 μm, Mg-Nd_I: R_a_ = 0.230 ± 0.008 μm, R_q_ = 0.369 ± 0.020 μm; Mg-Zn_R: R_a_ = 0.194 ± 0.019 μm, R_q_ = 0.264 ± 0.013 μm, Mg-Zn_I: R_a_ = 0.226 ± 0.019 μm, R_q_ = 0.337 ± 0.022 μm). These results are in good agreement with the contact angle and SFE values associated with a slightly rougher surface with a highly hydrophilic behavior for the Mg-Nd_I sample compared to the Mg-Zn_I sample. The beneficial effect of the fluoride coating is also evident in this profilometry analysis, as it increases roughness, thereby facilitating excellent cell adhesion to the implant surface.

To bridge the gap between topography and biology, the impact of roughness on cell attachment was quantified. The arithmetic average surface roughness R_a_ had values between 0.194 μm and 0.230 μm in the case of the investigated samples. These values generate “nanoniches”, which trap proteins such as fibronectin and vitronectin, preparing a favorable surface for cell adhesion. Quantitatively, the slightly higher roughness observed in the implant samples increases the effective surface area available for protein adsorption. Additionally, osteoblasts are linked to the implant or material via integrins, and the obtained root-mean-square roughness (R_q_) values provide physical points to clamp the integrin. The studies of Anselme et al. [113], Ponche et al. [114], and Deligianni et al. [115] showed that a surface characterized by a roughness near 0.2 μm sustains the focal adhesion for integrin compared to mirror-polished surfaces, being adequate for bone regeneration and characterized as presented in the next section by a good cell viability.

There are just a few studies that investigated the surface roughness of Mg-based alloys with fluoride conversion coatings. Merson et al. [116] investigated the improvement provided by HF treatment applied to the surface of Mg-2Zn-Ca-Al (ZX20) in terms of corrosion and stress corrosion cracking performance. The R_a_ roughness parameter increased after the surface treatment from 0.430 ± 0.010 μm (machined reference samples), 0.230 ± 0.020 μm (polished reference samples), to 0.840 ± 0.010 μm (machined HF-treated samples), and 0.400 ± 0.010 μm (polished HF-treated samples). The HF surface treatment consisted of immersing the sample in 40% HF for 15 min in an ultrasonic bath, followed by cleaning with ethanol, distilled water, and air drying. These results agree with those we obtained, and it can be concluded that HF treatment induced an increase in surface roughness. Rezaei-Baravati et al. [94] immersed Mg-Zn-Al-0.5Ca in 40% HF for 24 h. It was measured based on atomic force microscopy, a roughness increase (R_a_ parameter) from 0.271 μm (pristine sample) to 0.374 μm (6 h HF immersion), 0.362 μm (12 h HF immersion), 0.351 μm (18 h HF immersion), and 0.335 μm (24 h HF immersion). As in other studies, it can be concluded that MgF_2_ layer formation during the implant manufacturing stage increases surface roughness.

It is worth mentioning that the surface characterization was focused on the topographic and energetic properties of the MgF_2_ film obtained in the case of implant samples compared to reference ones. However, the bulk morphology of the reference samples has been previously presented via the SEM-EDS method [35], so in the current study, a research approach based on Surface Free Energy (SFE) computation and roughness measurement was applied. These quantitative findings are particularly important because they show the biological response of the cell lines to Mg-based implants. It is well known that osteoblast adhesion and protein docking are directly linked to the surface wettability and thermodynamic state. In addition, the MgF_2_ layer’s presence is indirectly confirmed by the significant shifts in contact angle and roughness values for the implant compared to the reference samples. In a previous investigation [35], the biocompatibility of the base alloys Mg-Nd and Mg-Zn was investigated based on the μ-CT method in a Sprague Dawley rat bone model. The in vivo findings could be considered a real proof-of-concept for material structural stability and bone regenerative potential. In conclusion, the CT data give a 3D image of the Mg-based alloy interaction with the living tissue and are very well complemented by the quantitative surface free energy computations obtained based on the OWKR model and roughness profile evolution.

### 3.3. Cytotoxicity Evaluation After Cell Culture Tests

The selected extract concentrations (250 μg/mL, 500 μg/mL, and 1000 μg/mL) were chosen to model the localized high-concentration environment resulting from the initial rapid degradation of magnesium-based implants. The maximum dose of 1000 μg/mL serves as a final test for cytocompatibility, following established protocols for evaluating the metabolic impact Mg^2+^ ions on osteoblasts [117,118,119].

**Figure 10:**
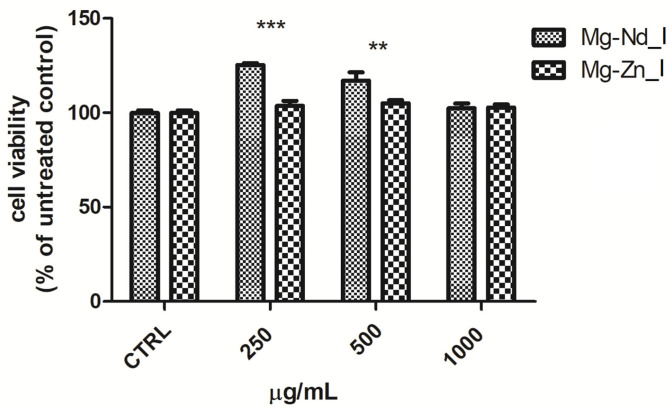
Viability of osteoblast cells after 24 h interaction with Mg-Nd_I and Mg-Zn_I implant extracts. ANOVA two-way, Bonferroni post-test (*n* = 3) (** *p* < 0.01, *** *p* < 0.001).

**Figure 11:**
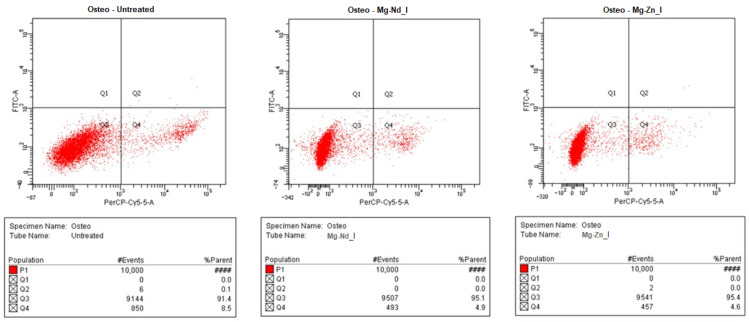
Flow cytometry analysis of osteoblast cells after 24 h exposure to Mg-Nd_I and Mg-Zn_I implant extracts cells at 1000 μg/mL. Osteoblast cells untreated (**left panel**) and treated with Mg-Nd_I implant extract (**middle panel**) and Mg-Zn_I implant extract (**right panel**), following annexin-FITC/PI staining. The viable cells (showing no apoptosis) were identified as Annexin V (−)/PI (−) (Q3). The apoptotic cells were identified as Annexin V (+)/PI (−) (early apoptosis) (Q1), and Annexin V (+)/PI (+) (late apoptosis) (Q2), while Annexin V (−)/PI (+) were considered necrotic cells (Q4).

Exposure of osteoblastic cells to the magnesium alloy implant extracts (Figure 10) did not adversely affect cell viability; moreover, the Mg–Nd_I sample at moderate concentrations enhanced cellular viability by 17% at 500 μg/mL (*p* < 0.01) and by 25% at 250 μg/mL (*p* < 0.001). To confirm these results, flow cytometric analysis was performed at the higher concentration of 1000 μg/mL. As shown in Figure 11, exposure to the magnesium alloy implants was not only non-toxic to osteoblastic cells but also appeared to exert a protective effect, reducing the proportion of necrotic cells from 8.6% in untreated control cells to 4.9% for the Mg–Nd_I implant extract and 4.6% for the Mg–Zn_I implant extract.

Both magnesium implant extracts showed no cytotoxicity; furthermore, the Mg–Nd_I sample significantly enhanced cell viability. Numerous studies have demonstrated that magnesium-containing substrates promote osteoblast proliferation in vitro. This stimulatory effect can be partly explained by improved cell–substrate interactions, most likely due to increased protein adsorption and strengthened integrin-mediated adhesion. Additionally, the release of Mg^2+^ ions may directly enhance osteoblast metabolic activity and proliferation by activating adhesion- and growth-associated signaling pathways. Consequently, the observed increase in osteoblast proliferation arises from a combination of surface-related effects and Mg^2+^-dependent biological mechanisms [120,121].

Annexin V–FITC/Propidium Iodide (PI) staining is a flow cytometry assay used to distinguish viable cells, early and late apoptosis, and necrosis based on membrane integrity and phosphatidylserine (PS) exposure. Annexin V binds phosphatidylserine (PS) in healthy cells, which is located on the inner leaflet of the plasma membrane. In early apoptotic cells, PS flips to the outer leaflet, allowing Annexin V binding, while propidium Iodide (PI) (a DNA-binding dye) cannot enter cells with an intact membrane. PI-positive cells lose the membrane integrity (late apoptosis or necrosis).

In the study, the addition of magnesium decreased the percentage of necrotic/non-viable cells. The soluble magnesium released from magnesium-based implant extracts to the cell culture medium can reduce the proportion of PI^+^/Annexin V–FITC^−^ cells, interpreted as primary necrotic cells. Magnesium ions play an important role in maintaining plasma membrane stability and cellular energy homeostasis, as Mg^2+^ is required for Adenosine Triphosphate (ATP)-dependent processes and mitochondrial function [120,121]. Adequate magnesium availability can therefore prevent acute energy failure and uncontrolled membrane rupture, favoring cell survival or the execution of regulated apoptotic pathways rather than necrosis. In addition, Mg^2+^ acts as a physiological antagonist of calcium, limiting calcium overload and the activation of membrane-degrading enzymes that contribute to necrotic cell death. Magnesium also reduces oxidative stress and lipid peroxidation, further protecting membrane integrity. Under conditions where magnesium release from the alloy is moderate and well buffered—without causing excessive alkalization, osmotic stress, or toxic accumulation of alloying elements—a decrease in PI^+^/Annexin V–FITC^−^ cells may be observed. In such cases, the reduced necrotic fraction may be accompanied by an increase in viable cells or a shift toward Annexin V-positive apoptotic populations, reflecting a transition from uncontrolled necrosis to more regulated cellular responses [122].

## 4. Conclusions

In this paper, it was developed an innovative manufacturing flow for Mg-based implants potentially used for bone defects. Attention was devoted to two Mg alloys produced by casting and used as primary materials for a previously described specifically designed bone implant. It was shown that the proposed production route is simple, based on biocompatible materials, and does not require expensive equipment with supplementary caution due to magnesium’s pyrophoric effects.

Compared to the additive manufacturing (AM) technique, especially with a focus on one of the most commonly used procedures in practice, such as selective laser melting (SLM), which is characterized by important industrial and biological drawbacks, the method developed in the paper solved these issues. Firstly, while the AM offers a wide range of model geometries, it is always followed by significant safety issues due to the pyrophoric effect of Mg-based powders. On the other hand, the hybrid weaving procedure eliminates the fire danger, making the presented manufacturing route much more scalable from an industrial point of view by reducing the costs of specialized explosion AM equipment. Direct AM is characterized by rapid melting and cooling cycles, which generate internal stresses and heat-affected zones that are usually linked to a premature failure of implants. In contrast, the presented casting-infiltration method permits a much slower and controlled cooling rate with important effects on implant corrosion and mechanical behavior. To end the list of advantages of the innovative developed method, it eliminates the so-called “staircase effect” characterized by the formation of a high roughness zone in a haphazard manner that can lead to a gas bubble trap or impact on the biological fluid flow. Regarding the wire-template approach, it generates cylindrical, smooth internal channels that are adequate for a laminar flow of the fluids and a uniform wall shear stress distribution as already described in [65].

It is well known that today there are some commercial alloys with chemical compositions similar to those analyzed in the paper, namely Mg-Zn-Mn (ZM21) and Mg-Zn-Zr (ZK60) for the Mg-Zn system, and Mg-Y-Nd-Zr (WE43), Mg-Y-Zn-Zr (WZ21), and Mg-Nd-Y-Zn-Zr (JDBM) for the Mg-Nd system. As documented in the literature and in line with already approved commercial products, the gold standard for orthopedic devices such as screws or implants remains the Mg-Y-Nd-Zr alloy, characterized by increased biocompatibility, high strength and ductility, and reduced weight loss. The Mg-Zn alloys pose a controversial challenge: the continuous need to search for an innovative elemental composition that balances corrosion rate and mechanical properties. In addition, it is important to note that Zn is an essential element for bone health. In our case, both Mg-based implants could be integrated into biological ranges with respect to their mechanical properties, weight loss, and biocompatibility, exhibiting excellent potential as future bone implants.

From a technical point of view, by analyzing mechanical suitability, one can immediately conclude that the implant samples exhibited superior behavior compared to the reference samples, as they minimized the stress-shielding effect. Implant samples had a compressive strength between 158 MPa and 188 MPa, much closer to that of human bone (131–205 MPa), a fact that proved an important design feature and the possibility of adjusting the implant’s mechanical properties to match those of natural bone. Regarding the Young’s modulus, the value of 16.79 ± 0.50 GPa obtained in the case of Mg-Nd_I sample is very similar to that of the trabecular bone (14.800 ± 1.40 GPa). In addition, the Mg-Zn_I (10.27 ± 0.7 MPa) elastic modulus is also favorable. As a direct consequence, the stress-shielding effect, defined as a critical factor for long-term implant success, does not appear in both cases. A tailored degradation profile was evident based on immersion tests. Mg-Zn_I sample (0.475 g weight loss) showed better structural integrity for a longer time when implanted in vivo conditions, compared with Mg-Nd_I sample (0.712 g weight loss). Implant samples presented high wettability, indicating an adequate surface energy for biological integration. Regarding the implant surface roughness, a slightly rougher surface was achieved (Mg-Nd_I: R_a_ = 0.230 μm versus Mg-Nd_R: R_a_ = 0.209 μm; Mg-Zn_I: R_a_ = 0.226 μm versus Mg-Zn_R: R_a_ = 0.194 μm).

Taking into consideration an important critical safety reason that is usually linked to Mg-based alloys, a corrosion byproduct, namely hydrogen gas (H_2_), can be dangerous. Firstly, it could lead to mechanical instabilities by generating gas pockets between the bone and the implant and preventing osteointegration. Then, a localized alkalinization (pH spike) could be observed, and if associated with pH values above 8.5, tissue necrosis is possible [117,118]; lastly, gas accumulation could induce pain or ischemia. In the framework of the study developed in the paper, the high cell viability obtained in the case of both investigated Mg-based implants confirmed that the protective MgF_2_ coating had a beneficial effect in reducing the amount of hydrogen emission as also underlined by Antoniac et al. [31].

Bone healing is a multicellular process that involves osteoblasts, osteoclasts, fibroblasts, and macrophage cells [123]. In the study presented here, the bone-building phase was analyzed exclusively, rather than the entire healing cycle of the bone defect. This approach has the following advantage: by involving only a single cell line (human patella-derived osteoblastic cells), it permits analysis of implant toxicity. Additionally, the paper’s goal is bone healing; the osteoblast cell line was correctly chosen, and it is well known that human primary cells are highly sensitive to pH changes, providing the necessary safety for in vitro investigations [32]. By using a single cell line, it was possible to see and understand the influence of surface morphology on cell proliferation without considering the complex intercellular signaling variables. In future studies, multiple cell lines will be added for a complete assessment of the bone healing process. In addition, the cytotoxic assessment was performed in triplicate; although this is commonly accepted for preliminary cytocompatibility assessments, additional replicates would strengthen the robustness of the results. Consequently, the present findings should be interpreted as osteoblastic compatibility rather than comprehensive biocompatibility, and additional in vitro models are necessary to better predict in vivo responses.

As a future direction, the in vivo behavior of the Mg-based implants will be investigated. In a previous study [35], a detailed in vivo analysis was conducted on Sprague Dawley rats involving Mg-Nd and Mg-Zn powders. Since non-toxic reactions were reported, it was concluded that the animal model tolerated the Mg-based powders, which demonstrated increased bone-regenerative potential. It is expected that the developed implants, which are characterized by a lower relative surface area and a much more controlled degradation profile, will perform better from a biological point of view. In addition, by considering in vitro conditions, it was shown that Mg-Nd implant increased osteoblast viability by up to 25% (*p* < 0.001) and reduced necrosis. In vivo medium exhibits a superior metabolic clearance and a higher buffering capacity compared to static cell culture, so it could be estimated that, in the case of an implant, a much more favorable biological response could be achieved. Another future direction that must undoubtedly be pursued is to extend the mechanical analysis to investigate the cyclic testing and dynamic and static loading conditions of the developed implants, and to compare them with the existing literature.

Some key “take-home” messages must be underlined. The first one should be formulated directly in terms of the thermodynamic stabilization of the surface. The SFE increased significantly during the MgF_2_ conversion coating formation step of the implant manufacturing process. This higher-energy state could be likened to an active surface that facilitates cell adhesion and protein adsorption. The second one is linked to the concept of preserving structural integrity. Lower weight losses were achieved for the implant samples, so the kinetic delay in the degradation profile indicates that the implant retains its mechanical properties during the critical first 14 days of bone healing. In this way, the implant failure could be avoided. The third “take-home” message concerns conformal topography, which is associated with improved surface uniformity. For instance, the contrast observed in the statistical analyses of R_a_ and R_q_ provides a nuanced view of the surface. R_a_ remains statistically similar (ns), whereas R_q_ shows a significant increase (**). This observation confirms that the MgF_2_ layer is conformal and ultra-thin, preserving the macrotopography of the implants while subtly altering the peak distributions (R_q_). This minor change can alter focal adhesion sites in osteoblasts without clogging the structure’s pores. The unintended, highly biocompatible MgF_2_ coating biofunctionalizes Mg-Nd and Mg-Zn implants, providing corrosion resistance, enhanced surface energy, and mechanical stability compared to the reference samples.

In conclusion, although Mg-Nd and Mg-Zn are highly promising for implant manufacturing, they remain in preclinical or clinical-stage trials. We described an original manufacturing procedure as an alternative to the current, expensive methods and an implant design that could be successfully used in future preclinical trials with optimal material and biological properties.

## Figures and Tables

**Table 1:** Nominal chemical composition of the two binary Mg-Nd and Mg-Zn alloys (wt%). (Note: Experimental validation via EDS for these specific alloys was previously reported in [35].)

Alloy	Composition (wt %)					
	Zn	Y	Nd	Ca	Mn	Mg
Mg-Nd	0.4	0.3	2.6	-	-	Bal.
Mg-Zn	1.4	-	-	0.3	0.6	Bal.

**Table 2:** Codification ID for implant and reference samples.

Mg-Based Alloy	Implant ID	Reference ID
Mg-Nd	Mg-Nd_I	Mg-Nd_R
Mg-Zn	Mg-Zn_I	Mg-Zn_R

**Table 3:** Printing parameters used for polymeric matrix manufacture.

Parameter	Value
Filament volume	5.08 cm^3^
Filament weight	6.30 g
Printing estimated time	5 h 21 min 12 s
Filament diameter	1.75 mm
Printing bed temperature	60 °C
Extruder diameter	0.2 mm
Printing speed	0.2 mm/s
Fill density	5%
Fill pattern	Gyroid

## Data Availability

The original contributions presented in the study are included in the article, further inquiries can be directed to the corresponding author.

## References

[1] Rahmati M., Mills D.K., Urbanska A.M., Saeb M.R., Venugopal J.R., Ramakrishna S., Mozafari M. (2021). Electrospinning for Tissue Engineering Applications. Prog. Mater. Sci..

[2] Bița T., Corneschi I., Ungureanu E., Bița A.-I., Ignat N.-D., Dura H., Cârstoc I.D. (2023). Influence of Heat Treatment on Microstructure and Corrosion Behavior of Biodegradable Mg-Ca Alloy. U.P.B. Sci. Bull. Ser. B.

[3] Pascu A., Oleksik V., Pirvu B., Fratila A., Ionas M., Boitor C. Modern Methods of Study and Research in Mechanical Engineering Applied to Medicine. Proceedings of the Balkan Region Conference on Engineering and Business Education & 2nd International Conference on Engineering and Business Education.

[4] Sharma P., Kumar P., Sharma R., Bhatt V.D., Dhot P. (2019). Tissue Engineering; Current Status & Futuristic Scope. J. Med. Life.

[5] Asadian M., Chan K.V., Norouzi M., Grande S., Cools P., Morent R., De Geyter N. (2020). Fabrication and Plasma Modification of Nanofibrous Tissue Engineering Scaffolds. Nanomaterials.

[6] Giannoudis P.V., Dinopoulos H., Tsiridis E. (2005). Bone Substitutes: An Update. Injury.

[7] Palmer S.H., Gibbons C.L.M.H., Athanasou N.A. (1999). The Pathology of Bone Allograft. J. Bone Jt. Surg. Br. Vol..

[8] Stratton S., Shelke N.B., Hoshino K., Rudraiah S., Kumbar S.G. (2016). Bioactive Polymeric Scaffolds for Tissue Engineering. Bioact. Mater..

[9] Raut H.K., Das R., Liu Z., Liu X., Ramakrishna S. (2020). Biocompatibility of Biomaterials for Tissue Regeneration or Replacement. Biotechnol. J..

[10] Hollister S.J., Maddox R.D., Taboas J.M. (2002). Optimal Design and Fabrication of Scaffolds to Mimic Tissue Properties and Satisfy Biological Constraints. Biomaterials.

[11] Liu Y., Nelson T., Chakroff J., Cromeens B., Johnson J., Lannutti J., Besner G.E. (2019). Comparison of Polyglycolic Acid, Polycaprolactone, and Collagen as Scaffolds for the Production of Tissue Engineered Intestine. J. Biomed. Mater. Res..

[12] Jazayeri H.E., Rodriguez-Romero M., Razavi M., Tahriri M., Ganjawalla K., Rasoulianboroujeni M., Malekoshoaraie M.H., Khoshroo K., Tayebi L. (2018). The Cross-Disciplinary Emergence of 3D Printed Bioceramic Scaffolds in Orthopedic Bioengineering. Ceram. Int..

[13] Lee S.S., Du X., Kim I., Ferguson S.J. (2022). Scaffolds for Bone-Tissue Engineering. Matter.

[14] Abbasian M., Massoumi B., Mohammad-Rezaei R., Samadian H., Jaymand M. (2019). Scaffolding Polymeric Biomaterials: Are Naturally Occurring Biological Macromolecules More Appropriate for Tissue Engineering?. Int. J. Biol. Macromol..

[15] Jin L., Li P., Wang Y.-C., Feng L., Xu R., Yang D.-B., Yao X.-H. (2019). Studies of Superb Microvascular Imaging and Contrast-Enhanced Ultrasonography in the Evaluation of Vascularization in Early Bone Regeneration. J. Ultrasound Med..

[16] Novosel E.C., Kleinhans C., Kluger P.J. (2011). Vascularization Is the Key Challenge in Tissue Engineering. Adv. Drug Deliv. Rev..

[17] Qiu L., Zhu Z., Peng F., Zhang C., Xie J., Zhou R., Zhang Y., Li M. (2022). Li-Doped Ti Surface for the Improvement of Osteointegration. ACS Omega.

[18] Guglielmotti M.B., Olmedo D.G., Cabrini R.L. (2019). Research on Implants and Osseointegration. Periodontology 2000.

[19] Antoniac I., Manescu (Paltanea) V., Paltanea G., Antoniac A., Fosca M., Laptoiu D., Rau J.V. (2025). Advancements in Biomaterials and Bioactive Solutions for Lumbar Spine Fusion Cages: Current Trends and Future Perspectives. Bioact. Mater..

[20] Xue N., Ding X., Huang R., Jiang R., Huang H., Pan X., Min W., Chen J., Duan J.-A., Liu P. (2022). Bone Tissue Engineering in the Treatment of Bone Defects. Pharmaceuticals.

[21] Filip N., Radu I., Veliceasa B., Filip C., Pertea M., Clim A., Pinzariu A.C., Drochioi I.C., Hilitanu R.L., Serban I.L. (2022). Biomaterials in Orthopedic Devices: Current Issues and Future Perspectives. Coatings.

[22] Nasrabadi H.N., Robu A., Ciocoiu R., Dumitrescu R., Necsulescu D.A., Fratila A.M. (2025). Evaluation of Surface Properties in 316L Stainless Steel Orthopedic Implants. U.P.B. Sci. Bull. Ser. B.

[23] Kharissova O.V., Méndez Y.P., Kharisov B.I., Nikolaev A.L., Dorozhkin S.V., Mena D.N., García B.O. (2025). Biomineralization of Calcium Phosphates in Nature. Nano-Struct. Nano-Objects.

[24] Varlik E., Viviant L., Kurtuldu F., Nawaz Q., Chen S., Kraxner J., Galusek D., Michálek M., Boccaccini A.R. (2026). Bioactive Glass (BG) Particle Shape Affects the Mechanical and Biological Properties of PLA/BG Scaffolds for Bone Regeneration. Mater. Lett..

[25] Moll M.N., Nawaz Q., Kunisch E., Ahrens T., Walker T., Renkawitz T., Boccaccini A.R., Westhauser F. (2025). Mesoporous Bioactive Glass Nanoparticles Exhibit Intrinsic Angiogenic Potential in the Chorioallantoic Membrane Assay, without the Addition of Exogenous Cells. Altern. Lab. Anim..

[26] Yu H., Liu H., Shen Y., Ao Q. (2023). Synthetic Biodegradable Polymer Materials in the Repair of Tumor-Associated Bone Defects. Front. Bioeng. Biotechnol..

[27] Farjaminejad S., Farjaminejad R., Hasani M., Garcia-Godoy F., Abdouss M., Marya A., Harsoputranto A., Jamilian A. (2024). Advances and Challenges in Polymer-Based Scaffolds for Bone Tissue Engineering: A Path Towards Personalized Regenerative Medicine. Polymers.

[28] Papuc A., Bran S., Moldovan M., Lucaciu O., Armencea G., Baciut G., Dinu C., Onișor F., Kretschmer W., Baciut M. (2024). How Is Bone Regeneration Influenced by Polymer Membranes? Insight into the Histological and Radiological Point of View in the Literature. Membranes.

[29] Antoniac I., Manescu (Paltanea) V., Paltanea G., Antoniac A., Nemoianu I.V., Petrescu M.I., Dura H., Bodog A.D. (2022). Additive Manufactured Magnesium-Based Scaffolds for Tissue Engineering. Materials.

[30] Antoniac I., Miculescu M., Mănescu (Păltânea) V., Stere A., Quan P.H., Păltânea G., Robu A., Earar K. (2022). Magnesium-Based Alloys Used in Orthopedic Surgery. Materials.

[31] Antoniac I., Manescu (Paltanea) V., Antoniac A., Paltanea G. (2023). Magnesium-Based Alloys with Adapted Interfaces for Bone Implants and Tissue Engineering. Regen. Biomater..

[32] Manescu (Paltanea) V., Antoniac I., Antoniac A., Laptoiu D., Paltanea G., Ciocoiu R., Nemoianu I.V., Gruionu L.G., Dura H. (2023). Bone Regeneration Induced by Patient-Adapted Mg Alloy-Based Scaffolds for Bone Defects: Present and Future Perspectives. Biomimetics.

[33] Dragomir (Nicolescu) L., Antoniac I., Manescu, Paltanea) V., Antoniac A., Miculescu M., Trante O., Streza A., Cotruț C.M., Forna D.A. (2023). Microstructure and Corrosion Behaviour of Mg-Ca and Mg-Zn-Ag Alloys for Biodegradable Hard Tissue Implants. Crystals.

[34] Kim Y.-K., Lee K.-B., Kim S.-Y., Bode K., Jang Y.-S., Kwon T.-Y., Jeon M.H., Lee M.-H. (2018). Gas Formation and Biological Effects of Biodegradable Magnesium in a Preclinical and Clinical Observation. Sci. Technol. Adv. Mater..

[35] Manescu (Paltanea) V., Antoniac A., Moraru M.C., Antoniac I., Cotrut C.M., Gradinaru S., Dreanca A.I., Sevastre B., Pop R., Tabaran F.A. (2025). Suitability of Mg-Nd and Mg-Zn Alloys to Obtain Biodegradable Structures for Bone Defects. JFB.

[36] (2025). Biological Evaluation of Medical Devices—Part 1: Requirements and General Principles for the Evaluation of Biological Safety Within a Risk Management Process.

[37] Ranakoti L., Gangil B., Bhandari P., Singh T., Sharma S., Singh J., Singh S. (2023). Promising Role of Polylactic Acid as an Ingenious Biomaterial in Scaffolds, Drug Delivery, Tissue Engineering, and Medical Implants: Research Developments, and Prospective Applications. Molecules.

[38] Maintz M., Tourbier C., de Wild M., Cattin P.C., Beyer M., Seiler D., Honigmann P., Sharma N., Thieringer F.M. (2024). Patient-Specific Implants Made of 3D Printed Bioresorbable Polymers at the Point-of-Care: Material, Technology, and Scope of Surgical Application. 3D Print. Med..

[39] da Silva D., Kaduri M., Poley M., Adir O., Krinsky N., Shainsky-Roitman J., Schroeder A. (2018). Biocompatibility, Biodegradation and Excretion of Polylactic Acid (PLA) in Medical Implants and Theranostic Systems. Chem. Eng. J..

[40] Drynda A., Hassel T., Hoehn R., Perz A., Bach F.-W., Peuster M. (2010). Development and Biocompatibility of a Novel Corrodible Fluoride-Coated Magnesium-Calcium Alloy with Improved Degradation Kinetics and Adequate Mechanical Properties for Cardiovascular Applications. J. Biomed. Mater. Res. A.

[41] Thomann M., Krause C., Angrisani N., Bormann D., Hassel T., Windhagen H., Meyer-Lindenberg A. (2010). Influence of a Magnesium-Fluoride Coating of Magnesium-Based Implants (MgCa0.8) on Degradation in a Rabbit Model. J. Biomed. Mater. Res. A.

[42] Yao W., Tan Y., Lu Q., Yi H., Cheng C., Wu L., Saji V.S., Pan F. (2024). Recent Advances in Protective Coatings and Surface Modifications for Corrosion Protection of Mg Alloys. J. Mater. Res. Technol..

[43] (2025). Standard Test Methods of Compression Testing of Metallic Materials at Room Temperature.

[44] Kokubo T., Kushitani H., Sakka S., Kitsugi T., Yamamuro T. (1990). Solutions Able to Reproduce in Vivo Surface-Structure Changes in Bioactive Glass-Ceramic A-W3. J. Biomed. Mater. Res..

[45] Zadpoor A.A. (2014). Relationship between in vitro Apatite-Forming Ability Measured Using Simulated Body Fluid and in vivo Bioactivity of Biomaterials. Mater. Sci. Eng. C.

[46] Pietrzyńska M., Voelkel A. (2017). Stability of Simulated Body Fluids Such as Blood Plasma, Artificial Urine and Artificial Saliva. Microchem. J..

[47] Kokubo T., Takadama H. (2006). How Useful Is SBF in Predicting in Vivo Bone Bioactivity?. Biomaterials.

[48] Standard Guide for Laboratory Immersion Corrosion Testing of Metals. https://store.astm.org/g0031-21r25.html.

[49] (2025). Standard Practice for Preparing, Cleaning, and Evaluating Corrosion Test Specimens.

[50] Zainal Abidin N.I., Atrens A.D., Martin D., Atrens A. (2011). Corrosion of High Purity Mg, Mg2Zn0.2Mn, ZE41 and AZ91 in Hank’s Solution at 37 °C. Corros. Sci..

[51] Cheng M., Wahafu T., Jiang G., Liu W., Qiao Y., Peng X., Cheng T., Zhang X., He G., Liu X. (2016). A Novel Open-Porous Magnesium Scaffold with Controllable Microstructures and Properties for Bone Regeneration. Sci. Rep..

[52] Zhang Z., Gupte M.J., Ma P.X. (2013). Biomaterials and Stem Cells for Tissue Engineering. Expert. Opin. Biol. Ther..

[53] Zhang B., Hou Y., Wang X., Wang Y., Geng L. (2011). Mechanical Properties, Degradation Performance and Cytotoxicity of Mg–Zn–Ca Biomedical Alloys with Different Compositions. Mater. Sci. Eng. C.

[54] Vojtěch D., Kubásek J., Čapek J. (2015). Comparative Mechanical and Corrosion Studies on Magnesium, Zinc and Iron Alloys as Biodegradable Metals. Mater. Tehnol..

[55] Annamalai M., Gopinadhan K., Han S.A., Saha S., Park H.J., Cho E.B., Kumar B., Patra A., Kim S.-W., Venkatesan T. (2016). Surface Energy and Wettability of van Der Waals Structures. Nanoscale.

[56] Keesom W.H. (1915). The Second Viral Coefficient for Rigid Spherical Molecules, Whose Mutual Attraction Is Equivalent to That of a Quadruplet Placed at Their Centre. Proceedings of the Royal Netherlands Academy of Arts and Sciences (KNAW) Proceedings.

[57] London F. (1937). The General Theory of Molecular Forces. Trans. Faraday Soc..

[58] Geometrical Product Specifications (GPS)—Surface Texture: Profile. https://www.iso.org/standard/72196.html.

[59] Tomuleasa C.I., Foris V., Soriţău O., Páll E., Fischer-Fodor E., Lung-Illes V., Brie I., Virág P., Perde-Schrepler M., Postescu I.D. (2009). Effects of 60Co Gamma-Rays on Human Osteoprogenitor Cells. Rom. J. Morphol. Embryol..

[60] Zhen Z., Liu X., Huang T., Xi T., Zheng Y. (2015). Hemolysis and Cytotoxicity Mechanisms of Biodegradable Magnesium and Its Alloys. Mater. Sci. Eng. C.

[61] Kim K.-J., Choi S., Sang Cho Y., Yang S.-J., Cho Y.-S., Kim K.K. (2017). Magnesium Ions Enhance Infiltration of Osteoblasts in Scaffolds via Increasing Cell Motility. J. Mater. Sci. Mater. Med..

[62] Mahmoud A., Ezgi Ö., Merve A., Özhan G. (2016). In Vitro Toxicological Assessment of Magnesium Oxide Nanoparticle Exposure in Several Mammalian Cell Types. Int. J. Toxicol..

[63] Di Virgilio A.L., Reigosa M., de Mele M.F.L. (2011). Biocompatibility of Magnesium Particles Evaluated by in Vitro Cytotoxicity and Genotoxicity Assays. J. Biomed. Mater. Res. B Appl. Biomater..

[64] (2009). Biological Evaluation of Medical Devices—Part 5: Tests for In Vitro Cytotoxicity. https://www.iso.org/standard/36406.html.

[65] Manescu Paltanea V., Paltanea G., Antoniac A., Gruionu L.G., Robu A., Vasilescu M., Laptoiu S.A., Bita A.I., Popa G.M., Cocosila A.L. (2024). Mechanical and Computational Fluid Dynamic Models for Magnesium-Based Implants. Materials.

[66] (2015). Computed Tomography in Dimensional Measurement—Determination of the Resolution of CT Systems.

[67] (2011). Computed Tomography in Dimensional Measurement—Guideline for the Application of Coordinate Measuring Systems with CT Sensors.

[68] (2019). Standard Guide for Computed Tomography (CT) Imaging.

[69] Drábiková J., Fintová S., Tkacz J., Doležal P., Wasserbauer J. (2017). Unconventional Fluoride Conversion Coating Preparation and Characterization. ACMM.

[70] Drynda A., Seibt J., Hassel T., Bach F.W., Peuster M. (2013). Biocompatibility of Fluoride-Coated Magnesium-Calcium Alloys with Optimized Degradation Kinetics in a Subcutaneous Mouse Model. J. Biomed. Mater. Res..

[71] da Conceicao T.F., Scharnagl N., Blawert C., Dietzel W., Kainer K.U. (2010). Surface Modification of Magnesium Alloy AZ31 by Hydrofluoric Acid Treatment and Its Effect on the Corrosion Behaviour. Thin Solid. Film..

[72] Yan T., Tan L., Zhang B., Yang K. (2014). Fluoride Conversion Coating on Biodegradable AZ31B Magnesium Alloy. J. Mater. Sci. Technol..

[73] Zhai C., Dai C.Y., Lv X., Shi B., Li Y.R., Yang Y., Fan D., Lee E.-S., Sun Y., Jiang H.B. (2022). Fluoride Coatings on Magnesium Alloy Implants. Bioinorg. Chem. Appl..

[74] Da Conceição T.F., Scharnagl N. (2015). Fluoride Conversion Coatings for Magnesium and Its Alloys for the Biological Environment. Surface Modification of Magnesium and Its Alloys for Biomedical Applications.

[75] Lou J., Sun Y., Chen Y., Zan R., Peng H., Yang S., Kang X., Peng Z., Wang W., Zhang X. (2021). Effects of MgF2 Coating on the Biodegradation and Biological Properties of Magnesium. Surf. Coat. Technol..

[76] Yazdimamaghani M., Razavi M., Vashaee D., Moharamzadeh K., Boccaccini A.R., Tayebi L. (2017). Porous Magnesium-Based Scaffolds for Tissue Engineering. Mater. Sci. Eng. C.

[77] Schmidt M., Waselau A.-C., Feichtner F., Julmi S., Klose C., Maier H.J., Wriggers P., Meyer-Lindenberg A. (2022). In Vivo Investigation of Open-Pored Magnesium Scaffolds LAE442 with Different Coatings in an Open Wedge Defect. J. Appl. Biomater. Funct. Mater..

[78] Seetharaman S., Sankaranarayanan D., Gupta M. (2023). Magnesium-Based Temporary Implants: Potential, Current Status, Applications, and Challenges. J. Funct. Biomater..

[79] Rynkus B., Scano A., Puxeddu S., Angius F., Ennas G., Szewczenko J. (2026). Biodegradable Magnesium Alloys for Short-Term Orthopedic Implants: Properties, Surface Modification and Biological Response. J. Mater. Res. Technol..

[80] Joshi P., Rao R.N., Golla C.B., Özcan M., Prasad P.S. (2025). Comprehensive Review of Mg-Based Alloys: Mechanical, Chemical and Biological Properties for Prosthetic and Orthopedic Applications. J. Mater. Res. Technol..

[81] Liu K., Zhang J., Su G., Tang D., Rokhlin L.L., Elkin F.M., Meng J. (2009). Influence of Zn Content on the Microstructure and Mechanical Properties of Extruded Mg–5Y–4Gd–0.4Zr Alloy. J. Alloys Compd..

[82] Prakash G., Singh N.K., Kumar D., Chandel P. (2020). Compression and Flexure Behaviours of Magnesium Alloy AZ41 at Different Strain Rates. U.P.B. Sci. Bull. Ser. D.

[83] Rho J.Y., Ashman R.B., Turner C.H. (1993). Young’s Modulus of Trabecular and Cortical Bone Material: Ultrasonic and Microtensile Measurements. J. Biomech..

[84] Kurniawan K.A., Wicaksono S.T., Rasyida A., Purniawan A. (2019). Improvement of Compressive Strength of Mg-Fe-Ca Alloy by Heat Treatment as Biodegradable Implant. Proceedings of the AIP Conference Proceedings.

[85] Teslia S., Kovalenko M., Teslia M., Vterkovskiy M., Solodkyi I., Loboda P., Soloviova T. (2024). The Activation of Magnesium Sintering by Zinc Addition. Alloys.

[86] Su Y., Lin J., Su Y., Zai W., Li G., Wen C. (2018). Investigation on Composition, Mechanical Properties, and Corrosion Resistance of Mg-0.5Ca-X(Sr, Zr, Sn) Biological Alloy. Scanning.

[87] Yilmaz B., Pazarceviren A.E., Tezcaner A., Evis Z. (2020). Historical Development of Simulated Body Fluids Used in Biomedical Applications: A Review. Microchem. J..

[88] Imani A., Rahimi E., Lekka M., Andreatta F., Magnan M., Gonzalez-Garcia Y., Mol A., Raman R.K.S., Fedrizzi L., Asselin E. (2024). Albumin Protein Impact on Early-Stage In Vitro Biodegradation of Magnesium Alloy (WE43). ACS Appl. Mater. Interfaces.

[89] Dong W., Matsukawa Y., Long Y., Hayashi Y., Nakamura J., Suzuki K., Ohtsuki C. (2024). Revised Method for Preparation of Simulated Body Fluid for Assessment of the Apatite-Forming Ability of Bioactive Materials: Proposal of Mixing Two Stock Solutions. RSC Adv..

[90] Pathak D.K., Pandey P.M. (2021). Evaluation of in Vitro Corrosion Behavior of Zinc–Hydroxyapatite and Zinc–Hydroxyapatite–Iron as Biodegradable Composites. J. Biomed. Mater. Res. Part B Appl. Biomater..

[91] Győri E., Fábián I., Lázár I. (2017). Effect of the Chemical Composition of Simulated Body Fluids on Aerogel-Based Bioactive Composites. J. Compos. Sci..

[92] Voicu M.-E., Golgovici F. (2020). A Biointerface Growth at Immersion of a Biodegradable Magnesium Alloy in Simulated Body Fluid. U.P.B. Sci. Bull. Ser. B.

[93] Bakhsheshi-Rad H.R., Idris M.H., Kadir M.R.A., Daroonparvar M. (2013). Effect of Fluoride Treatment on Corrosion Behavior of Mg–Ca Binary Alloy for Implant Application. Trans. Nonferrous Met. Soc. China.

[94] Rezaei-Baravati A., Kasiri-Asgarani M., Bakhsheshi-Rad H.R., Omidi M., Karamian E. (2022). Effect of Fluoride Coating on the Degradation of Mg-Based Alloy Containing Calcium for Biomedical Applications. J. Adv. Mater. Process..

[95] Ishizaki T., Saito N., Takai O. (2010). Correlation of Cell Adhesive Behaviors on Superhydrophobic, Superhydrophilic, and Micropatterned Superhydrophobic/Superhydrophilic Surfaces to Their Surface Chemistry. Langmuir.

[96] Silva G.A.F., Faot F., da Silva W.J., Del Bel Cury A.A. (2021). Does Implant Surface Hydrophilicity Influence the Maintenance of Surface Integrity after Insertion into Low-Density Artificial Bone?. Dent. Mater..

[97] Gianfreda F., Raffone C., Antonacci D., Mussano F., Genova T., Chinigò G., Canullo L., Bollero P. (2021). Early Biological Response of an Ultra-Hydrophilic Implant Surface Activated by Salts and Dry Technology: An In-Vitro Study. Appl. Sci..

[98] Wu S., Jang Y.-S., Lee M.-H. (2021). Enhancement of Bone Regeneration on Calcium-Phosphate-Coated Magnesium Mesh: Using the Rat Calvarial Model. Front. Bioeng. Biotechnol..

[99] Aronov D., Rosen R., Ron E.Z., Rosenman G. (2006). Tunable Hydroxyapatite Wettability: Effect on Adhesion of Biological Molecules. Process Biochem..

[100] Bita T., Antoniac A., Ciuca I., Miculescu M., Cotrut C.M., Paltanea G., Dura H., Corneschi I., Antoniac I., Carstoc I.D. (2023). Effect of Fluoride Coatings on the Corrosion Behavior of Mg–Zn–Ca–Mn Alloys for Medical Application. Materials.

[101] Quan P.H., Antoniac I., Miculescu F., Antoniac A., Manescu (Paltanea) V., Robu A., Bița A.-I., Miculescu M., Saceleanu A., Bodog A.D. (2022). Fluoride Treatment and In Vitro Corrosion Behavior of Mg-Nd-Y-Zn-Zr Alloys Type. Materials.

[102] Ashong A.N., Lee Y.S., Park K.S., Lee M., Kim J.H. (2021). Effect of HF Treatment on the Bonding Strength of Laser-Bonded Mg Alloy/Carbon Fiber-Reinforced Plastic Joint: XPS and NEXAFS Study. Appl. Surf. Sci..

[103] Xu W., Sato K., Koga Y., Sasaki M., Niidome T. (2020). Corrosion Resistance of HF-Treated Mg Alloy Stent Following Balloon Expansion and Its Improvement through Biodegradable Polymer Coating. J. Coat. Technol. Res..

[104] Zhao Y., Jamesh M.I., Li W.K., Wu G., Wang C., Zheng Y., Yeung K.W.K., Chu P.K. (2014). Enhanced Antimicrobial Properties, Cytocompatibility, and Corrosion Resistance of Plasma-Modified Biodegradable Magnesium Alloys. Acta Biomater..

[105] Höh N.V.D., Bormann D., Lucas A., Denkena B., Hackenbroich C., Meyer-Lindenberg A. (2009). Influence of Different Surface Machining Treatments of Magnesium-Based Resorbable Implants on the Degradation Behavior in Rabbits. Adv. Eng. Mater..

[106] Shetty O., Parekh R.B., Tabassum R. (2012). Surface Modifications for Endosseous Dental Implants. Int. J. Oral. Implantol. Clin. Res..

[107] Lacefield W.R. (1999). Materials Characteristics of Uncoated/Ceramic-Coated Implant Materials. Adv. Dent. Res..

[108] Lorenz C., Brunner J.G., Kollmannsberger P., Jaafar L., Fabry B., Virtanen S. (2009). Effect of Surface Pre-Treatments on Biocompatibility of Magnesium. Acta Biomater..

[109] Walter R., Kannan M.B., He Y., Sandham A. (2013). Effect of Surface Roughness on the in Vitro Degradation Behaviour of a Biodegradable Magnesium-Based Alloy. Appl. Surf. Sci..

[110] Laycock N.J., Krouse D.P., Hendy S.C., Williams D.E. (2014). Computer Simulation of Pitting Corrosion of Stainless Steels. Interface Mag..

[111] Packham D.E. (2003). Surface Energy, Surface Topography and Adhesion. Int. J. Adhes. Adhes..

[112] Baldan A. (2012). Adhesion Phenomena in Bonded Joints. Int. J. Adhes. Adhes..

[113] Anselme K., Ponche A., Bigerelle M. (2010). Relative Influence of Surface Topography and Surface Chemistry on Cell Response to Bone Implant Materials. Part 2: Biological Aspects. Proc. Inst. Mech. Eng. H.

[114] Ponche A., Bigerelle M., Anselme K. (2010). Relative Influence of Surface Topography and Surface Chemistry on Cell Response to Bone Implant Materials. Part 1: Physico-Chemical Effects. Proc. Inst. Mech. Eng. H.

[115] Deligianni D.D., Katsala N.D., Koutsoukos P.G., Missirlis Y.F. (2000). Effect of Surface Roughness of Hydroxyapatite on Human Bone Marrow Cell Adhesion, Proliferation, Differentiation and Detachment Strength. Biomaterials.

[116] Merson E.D., Poluyanov V.A., Myagkikh P.N., Bunev A.S., Merson D.L., Vinogradov A. (2023). Improving Corrosion and Stress Corrosion Cracking Performance of Machined Biodegradable Alloy ZX20 by HF-Treatment. Metals.

[117] Witte F., Kaese V., Haferkamp H., Switzer E., Meyer-Lindenberg A., Wirth C.J., Windhagen H. (2005). In Vivo Corrosion of Four Magnesium Alloys and the Associated Bone Response. Biomaterials.

[118] Witte F. (2010). The History of Biodegradable Magnesium Implants: A Review☆. Acta Biomater..

[119] Liu Y., Zheng Y., Chen X., Yang J., Pan H., Chen D., Wang L., Zhang J., Zhu D., Wu S. (2019). Fundamental Theory of Biodegradable Metals—Definition, Criteria, and Design. Adv. Funct. Mater..

[120] Zhu W., Wang W., Yang X., Ran C., Zhang T., Huang S., Yang J., Wang F., Wang H., Wan P. (2025). Research Progress on Osteoclast Regulation by Biodegradable Magnesium and Its Mechanism. Regen. Biomater..

[121] Long S., Wang W., Chen Y., Wang Z., Duan H., Yuan P., Xu Y., Li D., Zhang W., Wang W. (2025). E7 Peptide and Magnesium Oxide-Functionalized Coaxial Fibre Membranes Enhance the Recruitment of Bone Marrow Mesenchymal Stem Cells and Promote Bone Regeneration. BMC Biotechnol..

[122] Khalili M.A., Tamjid E. (2021). Controlled Biodegradation of Magnesium Alloy in Physiological Environment by Metal Organic Framework Nanocomposite Coatings. Sci. Rep..

[123] De Luca A., Ruggiero R., Cordaro A., Marrelli B., Raimondi L., Costa V., Bellavia D., Aiello E., Pavarini M., Piccininni A. (2024). Towards Accurate Biocompatibility: Rethinking Cytotoxicity Evaluation for Biodegradable Magnesium Alloys in Biomedical Applications. J. Funct. Biomater..

